# Plasma Desmosine Is Elevated in Thoracoabdominal Aortic Aneurysms and Is Associated with Intramural Proteolytic Activity

**DOI:** 10.3390/ijms27031236

**Published:** 2026-01-26

**Authors:** Panagiotis Doukas, Cathryn Bassett, Bernhard Hruschka, Elena Kuzmanova, Inga Wessels, Hannes J. Klump, Leon J. Schurgers, Michael J. Jacobs, Christian Uhl, Alexander Gombert, Jeffrey T. J. Huang

**Affiliations:** 1 Department of Vascular Surgery, RWTH Aachen University Hospital, 52074 Aachen, Germany; 2 Department of Biochemistry, Cardiovascular Research Institute Maastricht (CARIM), Maastricht University, 6200 MD Maastricht, The Netherlands; 3 Division of Systems Medicine, University of Dundee, Dundee DD1 9SY, UK; 4 Institute of Immunology, RWTH Aachen University, 52074 Aachen, Germany; 5 Center of Allergy and Environment (ZAUM), Technical University and Helmholtz Center, 80802 Munich, Germany; 6 Institute for Transfusion Medicine and Cell Therapeutics, RWTH Aachen University Hospital, 52074 Aachen, Germany

**Keywords:** aortic aneurysm, thoracoabdominal, desmosine, elastin metabolism, matrix metalloproteinases, biomarkers, blood, risk assessment

## Abstract

Thoracoabdominal aortic aneurysms (TAAAs) are rare and often remain asymptomatic until rupture, leading to high morbidity and mortality. Elastin degradation, largely mediated by matrix metalloproteinases (MMPs), plays a central role in their pathogenesis. This study aimed to evaluate plasma desmosine (pDES), a specific biomarker of elastin breakdown, as a non-invasive tool for TAAA detection and risk stratification. In a prospective single-centre case–control study, 30 patients with TAAA and 30 age- and sex-matched controls were enrolled. Plasma pDES levels were quantified using liquid chromatography–tandem mass spectrometry (LC–MS/MS). Aortic wall samples from 12 patients were analysed for elastic fibre content and MMP expression by histology and western blotting. Statistical analyses included correlation testing, propensity score matching, and receiver operating characteristic (ROC) analysis. TAAA patients exhibited significantly higher pDES levels compared with controls (0.40 ± 0.31 vs. 0.22 ± 0.15 ng/mL; *p* < 0.001). pDES correlated positively with MMP-2 (ρ = 0.68, *p* = 0.02), TIMP-1 (ρ = 0.72, *p* = 0.01), and the proportion of elastic fibres in the aortic media (ρ = 0.61, *p* = 0.03). ROC analysis showed good diagnostic performance (AUC = 0.82), with a threshold of 0.27 ng/mL yielding 78.6% sensitivity and 76.7% specificity. Elevated pDES levels reflect aortic elastolytic activity and may serve as a promising biomarker for TAAA detection and risk assessment.

## 1. Introduction

Thoracoabdominal aortic aneurysms (TAAAs), characterized by the progressive dilation of the aorta spanning the chest and abdomen, pose a significant clinical challenge due to their often silent progression and catastrophic risk of rupture. While relatively rare, affecting an estimated 5.9 per 100,000 person-years [1], the consequences of undiagnosed or untreated TAAAs are severe, leading to high morbidity and mortality.

Early detection is paramount, yet TAAAs are frequently discovered incidentally during imaging for unrelated conditions [2]. Current guidelines advocate for targeted screening, including imaging of first-degree relatives of patients with thoracic aortic disease [3] and extending CT angiography of the chest to individuals diagnosed with abdominal aortic aneurysms (AAAs), given their frequent co-occurrence [4]. However, the limitations of widespread imaging, such as radiation exposure and potential renal injury, underscore the need for non-invasive screening alternatives. Consequently, research efforts have focused on identifying non-invasive biomarkers or gene expression profiles as potential tools for TAAA screening [5]. While markers like D-dimer, matrix metalloproteinases (MMPs), and other acute-phase reactants have shown promise in symptomatic cases or in the presence of acute dissection, their utility for identifying asymptomatic patients remains limited [5].

The pathogenesis of TAAAs primarily involves the degradation of elastin in the aortic wall, driven by factors like ageing, hypertension [6], and genetic predispositions, including connective tissue disorders [7] and chronic aortic dissection [8]. The complex interplay of mechanical stress and inflammatory mediators can stimulate MMP production by both infiltrating immune cells and resident aortic cells [9]. MMP-mediated degradation of the tunica media leads to increased elastolysis and a loss of aortic wall distensibility [10]. This process contributes to aneurysm progression [11] and an increased risk of rupture. Notably, the upregulation of MMP-2 and MMP-9, along with a decrease in Tissue Inhibitor of Metalloproteinases 1 (TIMP-1)—resulting in an increase in the MMP/TIMP-1 ratio—has been recognized as a key event in aneurysmal growth [12]. Moreover, inhibiting macrophage elastase MMP-12 has been proposed as a potential therapeutic strategy to slow the development and progression of AAAs [13]. Thus, biomarkers associated with vascular elastolysis could serve as a promising monitoring screening tool for assessing aneurysm vulnerability [14].

In abdominal aortic aneurysms, serum elastin peptides [15] and plasma desmosine (pDES) [16], a specific marker of mature elastin breakdown, have shown promise as indicators of rupture risk. However, the applicability of these biomarkers to TAAAs remains largely unexplored. Given the expected cumulative elastolytic burden in TAAAs, circulating pDES may provide a measurable systemic signal reflective of this multiregional degradation process. This study aims to investigate the potential association of pDES levels and MMP-mediated elastolysis in the aortic wall of TAAA patients, evaluating the potential of pDES as a novel screening parameter for this high-risk disease condition.

## 2. Results

### 2.1. Cohort Description

The total patient cohort included 30 patients, with a mean age of 49.9 ± 11 years, and 20 (66.6%) were men. Eight patients (26.6%) were diagnosed with Marfan syndrome, and 17 (56.6%) had post-dissection aneurysms. Demographic details and their correlation with pDES levels are summarized in Table 1. The mean maximum aortic diameter was 6.1 ± 0.81 cm and showed no significant correlation with pDES levels (Spearman’s Rho = −0.14, *p* = 0.5). Men exhibited significantly higher pDES levels than women (*p* = 0.031; Supplementary Figure S1).

### 2.2. pDES and MMP Relative Intensity in the Aortic Wall

**Figure 1:**
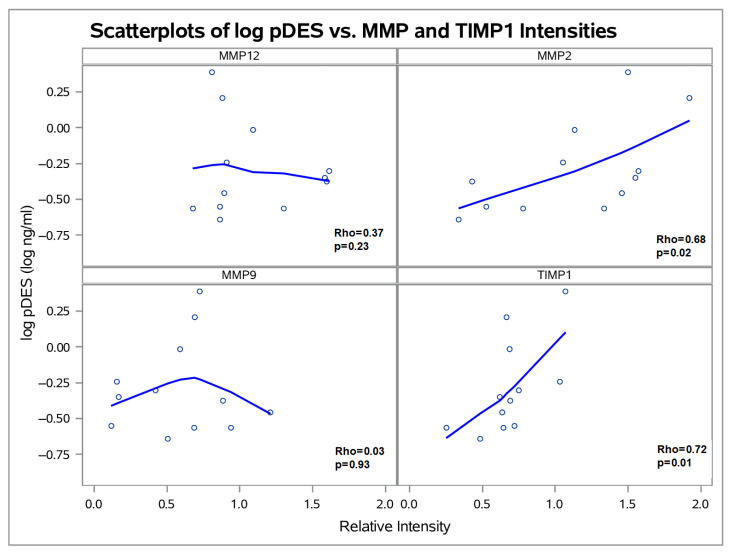
Scatterplots illustrating the relative intensity of aortic MMP-2, MMP-9, MMP-12, and TIMP-1 detected by Western Blot, plotted against log(pDES). A LOESS-smoothed best-fit line is included to highlight trends in the data.

Western blot analysis was performed in the subset of 12 TAAA patients from whom full-thickness aortic wall samples were available. In the Western Blot analysis of aortic tissue samples, the pDES levels were significantly correlated with the relative intensities of MMP-2 (Spearman’s Rho = 0.68, *p* = 0.02) and TIMP-1 (Spearman’s Rho = 0.72, *p* = 0.01) in the aortic wall (Figure 1). No significant correlations were identified for MMP-9 or MMP-12 and pDES. Additionally, circulating pDES levels positively correlated with percentage of elastic fibers in the aortic media (Spearman’s Rho = 0.61, *p* = 0.03; Supplementary Figure S2). The MMP-2/TIMP-1 ratio (mean: 1.76 ± 0.9) did not correlate with pDES levels (*p* = 0.65) (Supplementary Figure S3) or maximum aortic diameter (*p* = 0.9). However, patients with higher pDES levels showed elevated absolute levels of both MMP-2 and TIMP-1, as indicated by the correlation between the sum of their relative intensities and circulating pDES (Spearman’s Rho = 0.75, *p* = 0.004) (Supplementary Figure S4). Furthermore, a trend toward a positive correlation was observed between MMP-2 and TIMP-1 (Spearman’s Rho = 0.3, *p* = 0.5). No other inter-MMP interactions exhibited a similar trend.

A general linear model was used to examine whether gender, maximum aortic diameter, MMP2 relative intensity, and TIMP1 relative intensity predicted pDES levels. The overall model was statistically significant, (*p* = 0.003), and explained 91% of the variance in pDES levels (R^2^ = 0.91). When examining the individual predictors, only MMP2 (*p* = 0.003) and TIMP1 (*p* < 0.001) were significant, while gender (*p* = 0.75) and maximum aortic diameter (*p* = 0.19) did not have significant effects.

### 2.3. pDES Levels in Patients and Controls

**Figure 2:**
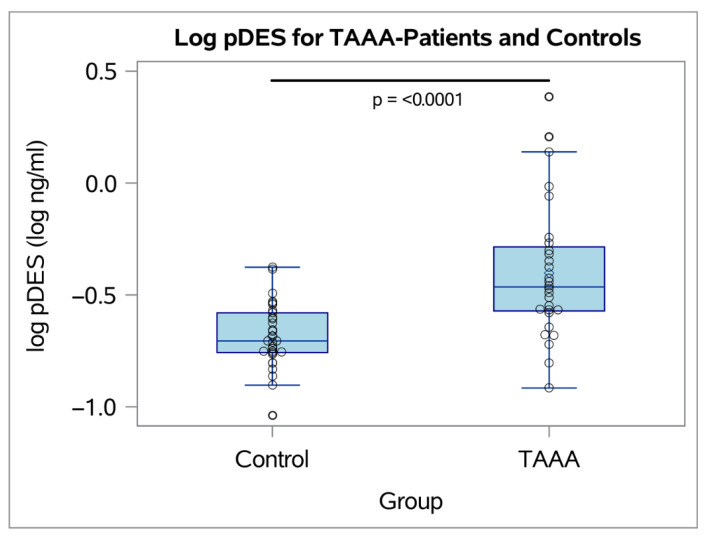
Boxplot showing the distribution of log-transformed pDES levels for TAAA and controls after propensity score matching.

After propensity score matching in the full cohort of 30 patients and 30 controls, 28 patients and all controls were included in the analysis. Matching reduced imbalances in covariates, with reductions in standardized mean differences for age (from 0.75 to 0.19, 74.73% reduction), logit propensity scores (from 0.80 to 0.18, 77.65% reduction), and gender (achieving perfect balance, standardized mean difference = 0). Mean log-transformed pDES levels were significantly higher in patients compared to controls (patients: −0.40 ± 0.31, controls: −0.68 ± 0.15; *p* < 0.001) (Figure 2).

### 2.4. Diagnostic Performance of pDES

**Figure 3:**
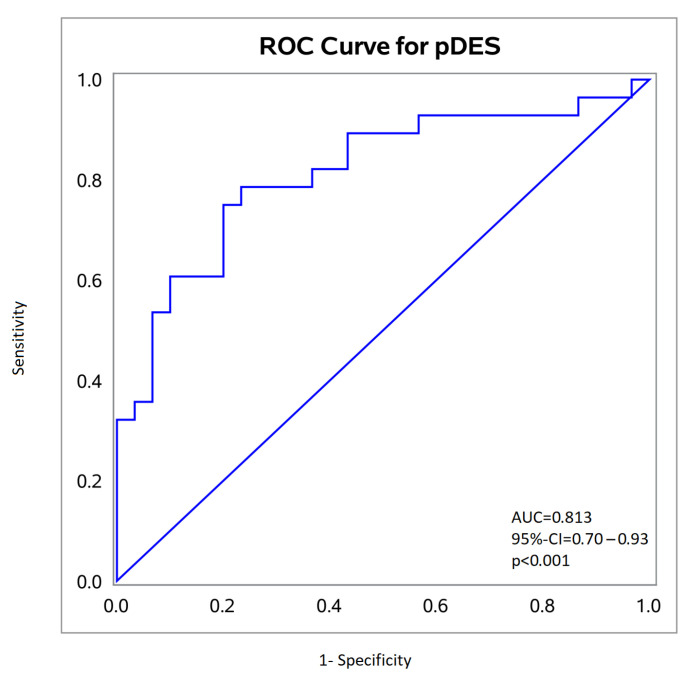
ROC Curve for pDES. AUC: Area under the curve.

ROC analysis demonstrated good diagnostic accuracy of pDES for TAAA, with an area under the curve (AUC) of 0.82 (*p* < 0.001) (Figure 3). The optimal cutoff was 0.27 ng/mL. At this threshold, sensitivity was 78.6%, specificity was 76.7%, and the Youden Index was 0.55.

## 3. Discussion

This study shows that circulating pDES can serve as a screening biomarker for TAAAs, and holds potential as novel diagnostic marker. Our key findings demonstrate a significant correlation between elevated pDES levels and increased proteolytic enzymatic activity, specifically MMP2 and TIMP1, within the aortic wall of TAAA patients. Notably, this correlation was independent of maximum aortic diameter—a suitable but not optimal marker for aortic aneurysm rupture risk. TAAA patients in this cohort exhibited significantly higher pDES levels compared to controls, with a diagnostic cut-off of 0.27 ng/mL.

Serum elastin peptides have been suggested with sufficient evidence as prognostic biomarkers for AAA growth and rupture [15,16] and correlated with serum concentrations of MMP2 and TIMP1 [16]. However, the expression of matrix metalloproteinases is not limited to the aortic wall and an elevation of their serum concentrations may reflect ECM remodelling processes in other segments of the vasculature [17] or even other organ systems [18,19], raising concerns about specificity. By directly measuring MMP expression within the aneurysmal aortic wall of TAAA patients, we confirmed a specific link between increased intramural proteolytic activity and elevated pDES levels in this patient group. To our knowledge, this aspect represents a novel area that has not been investigated to date.

Furthermore, pDES levels correlated directly with MMP2 and TIMP1 expression in the aortic wall, but not with aortic diameter—a finding which contrasts with previous studies that demonstrated a direct link between serum elastin peptides and aortic diameter in AAA patients [16,20]. This discrepancy may reflect cohort differences. Specifically, our TAAA patients were scheduled for elective surgical repair, meeting strict indication criteria based primarily on maximum aortic diameter [21]. Consequently, our cohort was relatively homogeneous regarding aortic size, with little variance of the parameter, unlike previous AAA studies, which included a wide range of aortic diameters [22]. Moreover, given that this study examined pathologies involving both the thoracic and abdominal aorta, the significantly higher elastin percentage in the thoracic aorta compared to the abdominal aorta could potentially influence the observed concentrations of elastolytic byproducts in serum.

Within this group of similarly large aneurysms, however, there was significant variation in aortic wall consistency, specifically in elastin content and the expression of MMPs and TIMP1. Elevated levels of both MMP and TIMP1 suggest ongoing proteolytic activity and extracellular matrix remodeling [23]. A disruption of the balance between MMP-mediated proteolysis and the regulatory action of TIMPs has been proposed as a mechanism driving aneurysm formation and progression [23]. In the presented cohort, increases in both TIMP1 and MMP2 levels correlated with higher pDES levels, whereas the ratio of MMP2 to TIMP1 did not correlate with pDES. This observation suggests that increased elastolysis may occur in aneurysms with a general upregulation of proteolytic activity, as reflected by elevated levels of both MMPs and compensatory TIMPs in the aortic wall, and is not solely attributable to an imbalance between these two protein fractions. The correlation of pDES levels with intramural proteolytic activity indicates that this biomarker could differentiate TAAAs based on proteolytic activity and thus support a more refined assessment of aneurysm prognosis. This finding provides an explanation and the biological backdrop of the observations of Mordi et al., who reported a strong association of pDES levels and adverse AAA events, such as rupture, independent of aortic diameter [16] and highlights the notion that aortic diameter alone might not suffice as a rupture risk stratification tool and intervention guide for AAA [24] and TAAA alike.

Understanding pDES as a marker of aortic wall elastolysis, our study found that only MMP2 and TIMP1 exhibited a clear correlation. Although previous research has implicated MMP9 and MMP12 in the formation and expansion of AAA [25], no significant relationship between these MMPs and pDES was observed in our TAAA cohort. MMP9 and MMP12 are primarily produced by inflammatory cells [26], and are central to AAA pathogenesis, whereas degenerative processes predominate in the descending thoracic aorta [27]. The lack of a conclusive association between pDES and these MMPs likely reflects the complex interplay among MMPs [28] and the variable impact of inflammation on elastolysis within this multisegmented pathology.

Considering its clinical potential, measuring pDES offers a promising alternative to a vast array of other biomarkers for AAA growth and rupture [15] and it has been reported that AAA patients display higher levels of pDES in comparison to healthy controls [16]. As previously speculated [29], serum elastin peptides may also serve as a diagnostic tool for thoracic aortic aneurysms—a silent disease in urgent need of effective screening. Beyond aneurysmal disease, the measurement of pDES may have further applications in the evaluation of acute aortic syndromes, including acute type B dissection, acute type A dissection, intramural hematoma and contained rupture. In these conditions, increased elastin degradation and extracellular matrix remodeling play a critical role in disease progression and wall instability. Monitoring pDES levels could potentially aid in identifying patients at higher risk of adverse events, support early diagnosis, and guide therapeutic decision-making.

A key novel finding of our study is the marked elevation of pDES in TAAA patients compared to healthy, age- and gender-matched controls. A pDES threshold of 0.27 ng/mL was able to distinguish TAAA patients from controls with a sensitivity of 78.6% and a specificity of 76.7%. It is important to note that this cutoff, derived from a relatively small cohort, may not be directly comparable to thresholds previously reported for AAA patients [16]. Although a direct comparison of AAA and TAAA patients is beyond the scope of this study, we speculate that the proteolytic degradation of the elastin-rich thoracic aorta, a process particularly prominent in TAAA [30], may significantly influence pDES levels. In support of this hypothesis, the mean percentage of elastic fibers in the aortic media within our cohort was directly proportional to and significantly correlated with pDES. Accordingly, we do not propose pDES as a biomarker exclusive to TAAAs, but as a biologically plausible and quantifiable indicator of the extensive elastolysis occurring in this multi-segmented disease. Whether pDES differs across isolated thoracic, isolated abdominal, and combined thoracoabdominal aneurysm disease remains unknown and warrants future investigation in dedicated comparative cohorts.

The findings of this study should be interpreted in the context of its limitations. First, although the cohort represents one of the largest series with paired plasma and full-thickness aortic tissue samples in TAAA patients, the sample size remains limited. This reflects both the rarity of TAAA and the restricted availability of surgically accessible tissue. As a consequence, replication in an independent cohort was not feasible and should be pursued in larger, multicenter studies. The small sample size may have reduced the statistical power of our analysis, potentially obscuring subtle patterns in the data. Although the primary endpoints were statistically supported, the relationships between MMP9, MMP12, and pDES remained inconclusive. Additionally, our immunohistological analysis focused on the most prominent enzymes involved in aortic elastolysis, thereby excluding a broader range of enzymes known to contribute to this process. We acknowledge that Western blot band visibility varied between samples, reflecting differences in tissue quality and protein yield. All quantitative analyses relied on normalized densitometric data rather than visual inspection. Moreover, as is common in studies of the human thoracoabdominal aorta, we were unable to include a comparable control cohort of healthy histological tissue specimens for assessing intramural proteolytic activity in the non-aneurysmatic aorta. Similarly, while our surgical tissue samples were meticulously dissected to include all three layers of the aortic wall, they represent only a portion of the diseased aorta and do not capture the full extent of intramural proteolytic activity. This limitation is particularly relevant in post dissection aneurysms, where the expansion of the aortic diameter is affected by the degenerative changes in the false lumen, potentially further influenced by enzymatic activity from the intraluminal thrombus, as observed in AAA [31]. Yet, in each case a tissue sample including the whole aortic wall could be gained. To minimize external variability, all tissue samples were collected and processed using a standardized protocol, ensuring a homogeneous cohort.

## 4. Materials and Methods

### 4.1. Study Design and Patient Cohorts

This single-centre, prospective, observational trial was conducted in compliance with the Declaration of Helsinki and the STROBE guidelines. The study protocol was reviewed and approved by the Ethics Committee of the University Hospital Aachen (EK010/19 and EK102/20). This study was registered in the German Clinical Trials Register (DRKS) under the registration number DRKS00036920. The study population consisted of two groups: patients undergoing elective open TAAA repair and a control group of healthy individuals. Control subjects were recruited during blood donation sessions. All control participants underwent routine screening to rule out underlying conditions, including aortic aneurysms and lung disease. In total, 30 patients with TAAA and 30 controls were included in the analysis. Written informed consent was obtained from all participants prior to their inclusion. Pregnant individuals and those under 18 years of age were excluded from the study. Data on patients’ medical history and demographic details were collected from digital medical records and clinical charts. Chronic obstructive pulmonary disease was diagnosed in patients with a post-bronchodilator FEV1/FVC ratio  <  0.7 [32]. Coronary artery disease was diagnosed in patients with chronic angina syndromes—with or without previous myocardial infarction or revascularization—and ischemic heart disease diagnosed by noninvasive testing [33].

### 4.2. Material Acquisition and Processing

Blood samples from patients were collected prior to surgery, while control samples were obtained during routine blood donation sessions. Intraoperative aortic tissue samples were obtained from 12 patients, each from at least one of three anatomical regions: the distal thoracic, suprarenal, or infrarenal aorta. Tissue resection was performed in a sterile environment, focusing exclusively on diseased segments of the aorta. Care was taken to ensure that all three layers of the aortic wall were included. Full-thickness samples could be obtained in 12 patients: these were the only specimens eligible for Western blot analysis. The availability of tissues was determined solely by intraoperative feasibility and patient safety. All tissue samples meeting the requirement of containing all three layers of the aortic wall were processed. Each sample was assigned an anonymous study identifier, which was carried through all analyses. In several patients, full-thickness aortic wall tissue could be harvested from more than one anatomical region (thoracic, suprarenal, infrarenal). Because intraoperative feasibility varied, the number of available segments differed between patients. Western blot analysis was therefore performed per anatomical segment, not per patient. For each patient, the densitometric intensities of all available segments were subsequently aggregated into a single per-patient value (mean relative intensity) for statistical correlation with plasma pDES. This aggregation approach was selected because each segment originates from the same aneurysm and reflects the patient-specific proteolytic profile. The anatomical origin and the aggregated per-patient values, are provided in Supplementary Table S1.

For histological analysis, the collected samples were preserved in a formaldehyde solution (3.5–4%), embedded in paraffin, and sectioned into 2-micrometer slices using a Leica SM200R microtome (Leica Biosystems Inc., Nußloch, Germany). The sections underwent MOVAT-pentachrome staining and were digitized at 20× magnification using a FRITZ Slidescanner (PreciPoint GmbH, Munich, Germany) at the Interdisciplinary Center for Clinical Research (IZKF) at University Hospital Aachen. Elastin content of vascular samples was assessed using the open-source software QuPath v0.5.1 (https://qupath.github.io/) [34]. The medial layer of each section was delineated, and a colour threshold was applied to identify elastic fibers, quantifying the elastin staining as a percentage relative to the medial layer. Artifacts and folds present in the tissue sections were manually excluded.

For Western blot analysis, tissue samples were lysed in 200 µL β-mercaptoethanol-containing lysis buffer and mechanically homogenized to release proteins. Insoluble debris was removed by centrifugation at 15,000 rpm for 1:30 min, and the supernatant containing soluble proteins was collected. Protein concentrations were measured using the BCA assay according to the manufacturer’s protocol [35]. Samples were boiled for 5 min at 95 °C and separated using 10% or 15% SDS-PAGE electrophoresis with 170 mV per gel. Proteins were then transferred onto a nitrocellulose membrane. The transfer process was carried out at 100 mV for 1 h. Following the transfer, the membranes were blocked for 30 min with 4% skimmed milk in TBS-T (0.1% Tween 20 in Tris-buffered saline). Membranes were incubated overnight at 4 °C with primary antibodies under gentle agitation. After incubation, membranes were washed three times for 10 min each with TBS-T and then exposed to a secondary antibody (diluted 1:10,000) for 1 h. The washing steps were repeated, and immunoreactive proteins were visualized using an enhanced chemiluminescence detection system. Band intensity was quantified densitometrically and normalized to β-actin levels using ImageJ Fiji (Release 2.17.0) [36].

pDES concentrations were measured using a validated stable isotope dilution liquid chromatography–tandem mass spectrometry method described previously [37].

### 4.3. Statistical Analysis

Continuous variables are presented as mean ± standard deviation (SD) or as median with interquartile range (IQR) for skewed data, while categorical variables are expressed as counts and percentages. Since pDES concentrations did not follow a normal distribution, they were log-transformed prior to analysis. Correlations were analyzed with the Spearman’s test. Propensity Score Matching was performed to balance observed covariates between patients and controls. Propensity scores were estimated using a logistic regression model, including age and gender as predictors. Patients and controls were matched using a greedy nearest-neighbor algorithm (1:1 ratio) with a calliper width of 0.5 on the logit of the propensity score. Matching was restricted to observations within the region of common support to ensure comparability, and exact matching was applied on gender. Balance was assessed using standardized mean differences and variance ratios. Matching improved the balance of covariates, with standardized mean differences for age and logit propensity scores reduced to acceptable levels (<0.25). Gender achieved perfect balance due to exact matching. A two-sample *t*-test was conducted on the matched dataset to compare log-transformed pDES levels between patients and controls. Receiver operating characteristic (ROC) curve analysis was conducted to evaluate the diagnostic potential of pDES for TAAA, with the optimal cut-off determined using the Youden index. All statistical tests were two-tailed, with significance set at *p* < 0.05. Analyses were performed using SAS software version 9.4 (© 2024 SAS Institute Inc., Cary, NC, USA).

## 5. Conclusions

pDES levels are significantly elevated in TAAA patients compared with a matched cohort of non-aneurysmatic patients. pDES correlates significantly with increased intramural MMP-mediated elastolysis—independent of aortic diameter—suggesting that pDES can serve as an indicator of aneurysms with active elastolytic processes. Given its promise as both a screening tool and a risk stratification adjunct for TAAA patients, larger studies are warranted to validate its clinical utility, especially in fields of acute aortic pathologies and progressive aneurysmal degeneration of the aorta in clinical asymptomatic patients.

## Figures and Tables

**Table 1:** Demographic details and correlations with pDES. Correlations for continuous variables were calculated with Spearman’s test and for categorical variables with Wilcoxon Two-Sample test. Asterisk (*) marks *p* < 0.05.

	*n* = 30	*p*-Value
Age, mean ± SD (years)	49.9 ± 11 years	0.97
Men, *n* (%)	20 (66.6)	0.031 *
Marfan syndrome, *n* (%)	8 (26.6)	0.83
Post-dissection aneurysm, *n* (%)	17 (56.6)	0.16
Max. diameter, mean ± SD (cm)	6.1 ± 0.81	0.31
Smoking	7 (23.3)	0.14
Chronic Obstructive Pulmonary Disease	6 (20)	0.17
Coronary Artery Disease	2 (0.6)	0.88

## Data Availability

The datasets generated and analysed during the current study are available from the corresponding author upon reasonable request. Due to ethical and privacy considerations, individual patient-level data cannot be shared publicly. The analytical methods (statistical code in SAS) and study materials are available upon request for the purpose of reproducing results or replicating procedures.

## Supplementary Materials

The following supporting information can be downloaded at: https://www.mdpi.com/article/10.3390/ijms27031236/s1. Representative histological samples and western blot images are shown in the Supplementary Materials.

## Abbreviations

The following abbreviations are used in this manuscript:

AAA	Abdominal Aortic Aneurysm
AUC	Area Under the Curve
DRKS	Deutsches Register Klinischer Studien (German Clinical Trials Register)
ECM	Extracellular Matrix
FEV1	Forced Expiratory Volume in 1 Second
FVC	Forced Vital Capacity
IZKF	Interdisciplinary Center for Clinical Research
LC–MS/MS	Liquid Chromatography–Tandem Mass Spectrometry
LOESS	Locally Estimated Scatterplot Smoothing
MMP	Matrix Metalloproteinase
pDES	Plasma Desmosine
ROC	Receiver Operating Characteristic
SAS	Statistical Analysis System
SD	Standard Deviation
STROBE	Strengthening the Reporting of Observational Studies in Epidemiology
TAAA	Thoracoabdominal Aortic Aneurysm
TIMP	Tissue Inhibitor of Metalloproteinases
