# Supplementary material for: Plasma Desmosine Is Elevated in Thoracoabdominal Aortic Aneurysms and Is Associated with Intramural Proteolytic Activity
Source: Int J Mol Sci. 2026 Jan 26;27(3):1236. doi: 10.3390/ijms27031236 (PMC12898253; doi:10.3390/ijms27031236)
Supplement: Supplementary file 1 [file ijms-27-01236-s001.zip › Supplementary Figures and Tables.pdf]

Plasma Desmosine Is Elevated in Thoracoabdominal Aortic Aneurysms and Is Associated with Intramural Proteolytic Activity

Panagiotis Doukas [1,2,*], Cathryn Bassett [1], Bernhard Hruschka [1], Elena Kuzmanova [3], Inga Wessels [4,5], Hannes J. Klump [6], Leon J. Schurgers [2], Michael J. Jacobs [1], Christian Uhl [1], Alexander Gombert [1,†] and Jeffrey T. J. Huang [3,†]

1 Department of Vascular Surgery, RWTH Aachen University Hospital, 52074 Aachen, Germany
2 Department of Biochemistry, Cardiovascular Research Institute Maastricht (CARIM), Maastricht University, 6200 MD Maastricht, The Netherlands
3 Division of Systems Medicine, University of Dundee, Dundee DD1 9SY, UK
4 Institute of Immunology, RWTH Aachen University, 52074 Aachen, Germany
5 Center of Allergy and Environment (ZAUM), Technical University and Helmholtz Center, 80802 Munich, Germany
6 Institute for Transfusion Medicine and Cell Therapeutics, RWTH Aachen University Hospital, 52074 Aachen, Germany
* Correspondence: pdoukas@ukaachen.de, Tel.: +49-241-803-6070
† These authors contributed equally to this work.

Supplementary Materials - Index

Supplementary Figures and Tables

Supplementary Figure S1 *pag. 2*
Supplementary Figure S2 *pag. 3*
Supplementary Figure S3 *pag. 4*
Supplementary Figure S4 *pag. 5*
Supplementary Table S1 *pag. 6*

Supplementary Figures and Tables

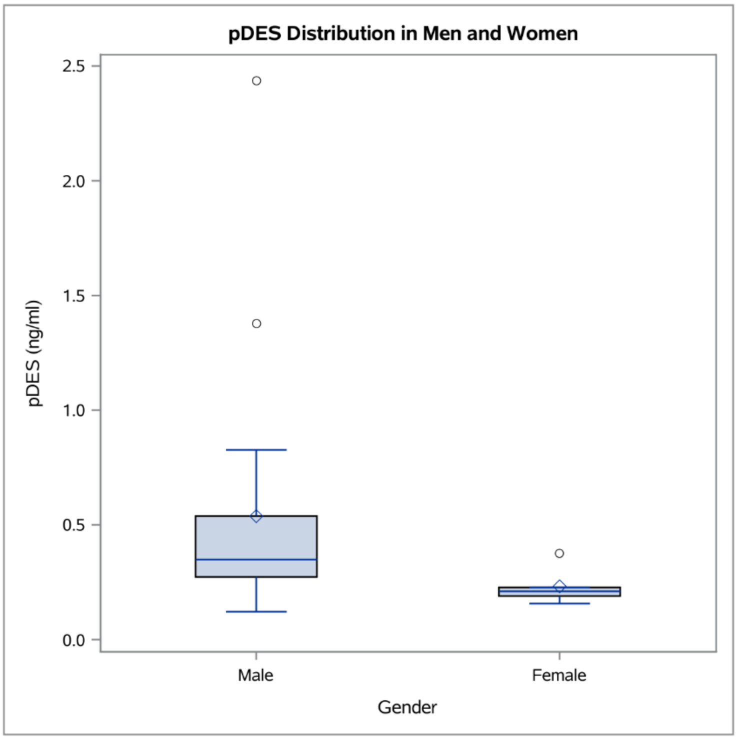

Supplementary Figure S1. Boxplot showing the distribution of pDES levels in male and female TAAA patients.

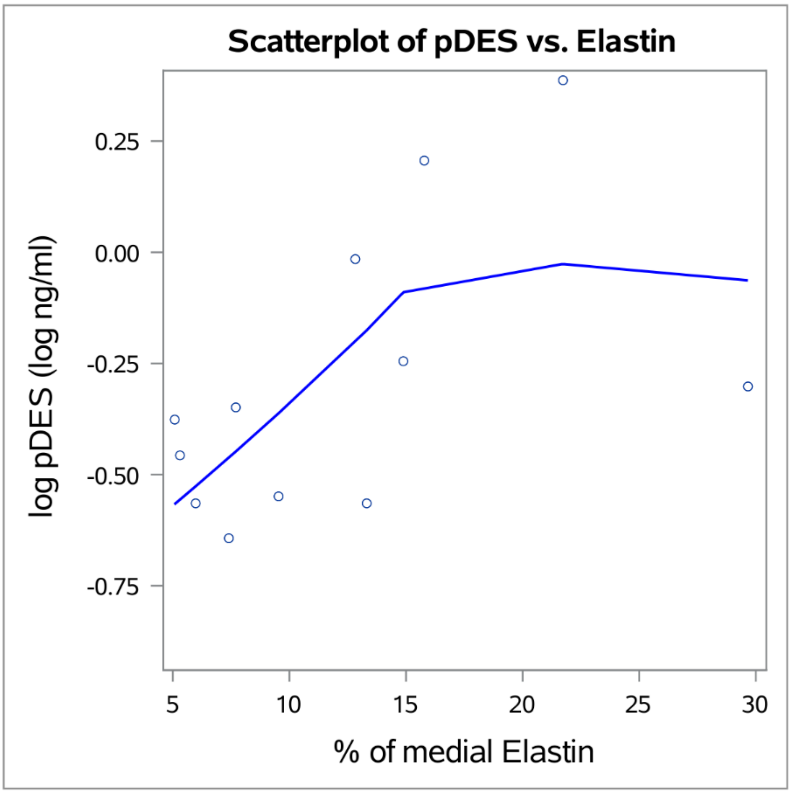

Supplementary Figure S2. Scatterplot illustrating of elastin percentage in the aortic media plotted against log(pDES). A LOESS-smoothed best-fit line is included to highlight trends in the data.

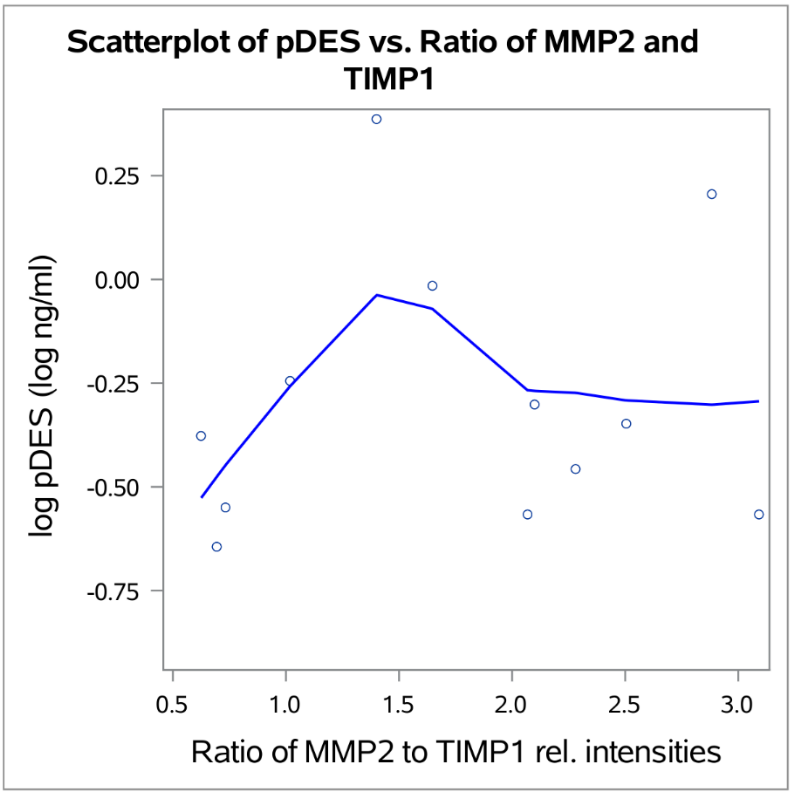

Supplementary Figure S3. Scatterplot illustrating the ratio of MMP2 and TIMP1 relative intensities plotted against log(pDES). A LOESS-smoothed best-fit line is included to highlight trends in the data.

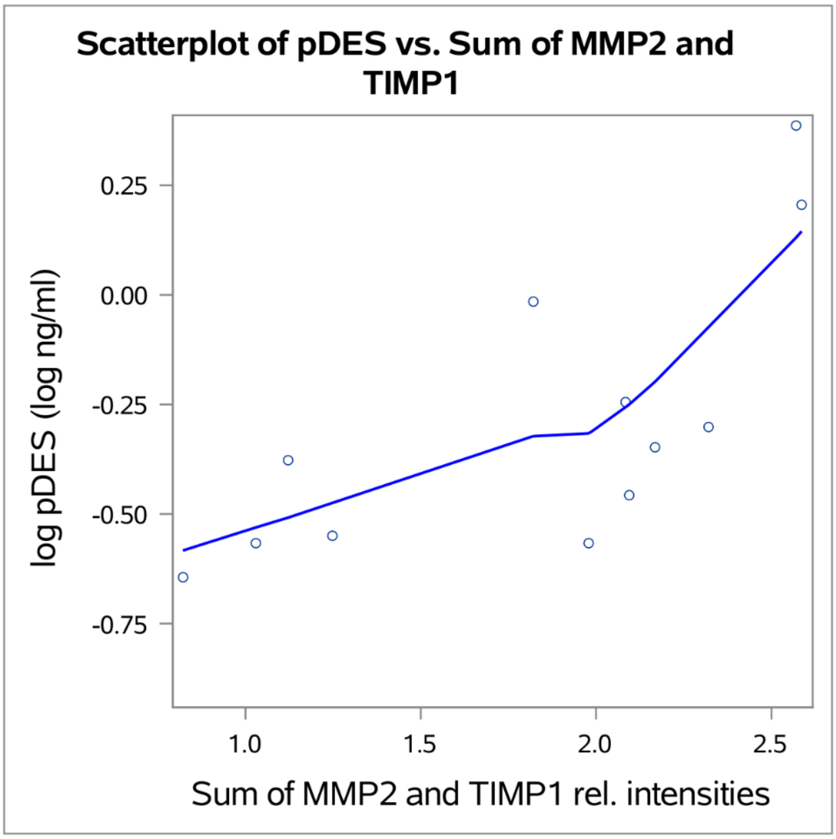

Supplementary Figure S4. Scatterplot illustrating the sum of MMP2 and TIMP1 relative intensities plotted against log(pDES). A LOESS-smoothed best-fit line is included to highlight trends in the data.

Supplementary Table S1. Anonymized patient identifiers, number and anatomical origin of full-thickness aortic wall segments used for Western blot analysis, corresponding plasma desmosine (pDES) values, and aggregated per-patient densitometric intensities of MMP2, MMP9, MMP12, and TIMP1.

**ID**	**Segments (n)**	**Types**	**pDES (ng/mL)**	**log(pDES)**	**MMP2**	**MMP9**	**MMP12**	**TIMP1**
**1**	3	T, SR, IR	0.50	−0.57	0.94	0.94	0.68	0.25
**2**	1	T	0.28	−0.64	0.51	0.51	0.87	0.48
**3**	1	T	0.42	−0.35	0.17	1.59	0.62	0.62
**4**	3	T, SR, IR	0.38	−0.46	1.21	0.89	0.64	0.64
**5**	2	SR, IR	0.35	−0.57	0.69	1.30	0.65	0.65
**6**	1	T	0.48	0.21	0.69	0.88	0.67	0.67
**7**	2	T, SR	0.19	−0.38	0.88	1.59	0.69	0.69
**8**	1	IR	0.16	−0.55	0.12	0.86	0.72	0.72
**9**	1	T	0.27	−0.30	0.42	1.61	0.75	0.75
**10**	2	T,SR	0.21	−0.24	0.15	0.91	1.03	1.03
**11**	1	T	0.21	0.39	0.73	0.81	1.07	1.07
**12**	1	SR	0.27	−0.02	0.59	0.59	1.09	0.69

Abbreviations: T = thoracic; SR = suprarenal; IR = infrarenal.
