# Supplementary material for: Plasma Desmosine Is Elevated in Thoracoabdominal Aortic Aneurysms and Is Associated with Intramural Proteolytic Activity
Source: Int J Mol Sci. 2026 Jan 26;27(3):1236. doi: 10.3390/ijms27031236 (PMC12898253; doi:10.3390/ijms27031236)
Supplement: Supplementary file 1 [file ijms-27-01236-s001.zip › raw data.pdf]

Representative Histological Samples

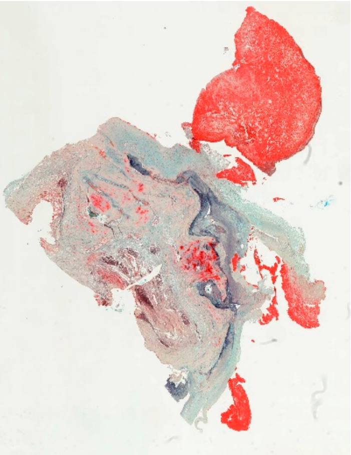

sample 001-I

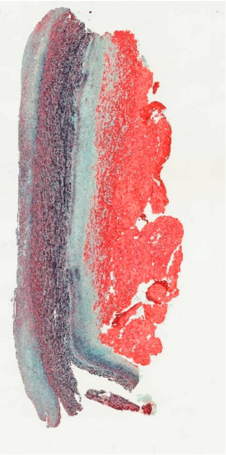

Sample 001-II

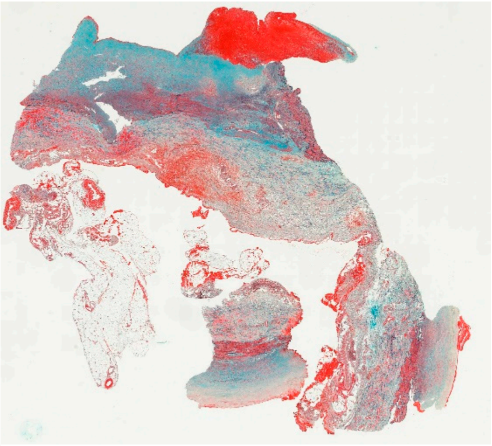

Sample 003-III

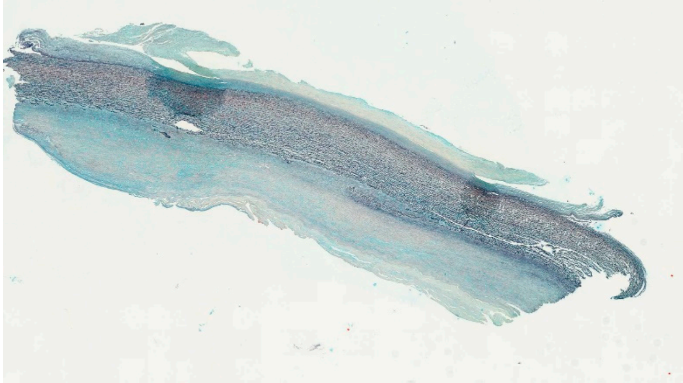
Sample 006-I

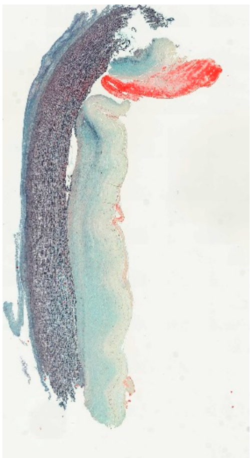

Sample 006-III

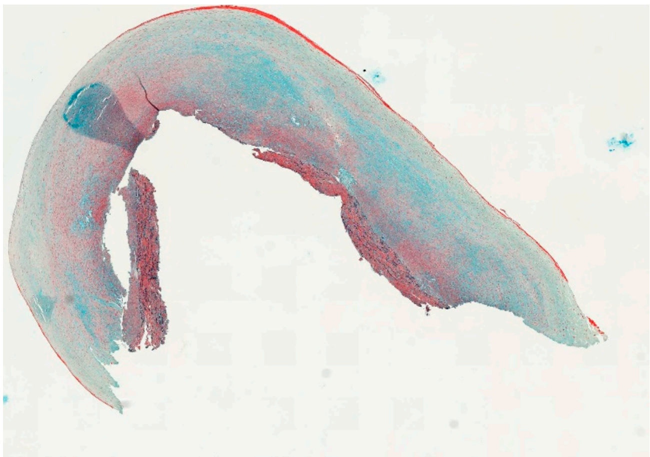
Sample 007-II

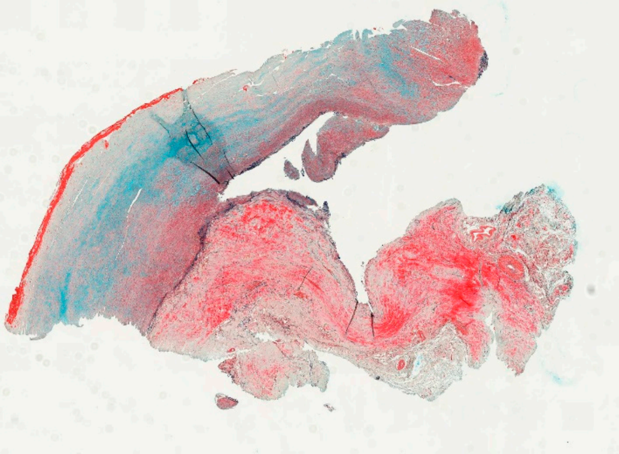
Sample 007-III

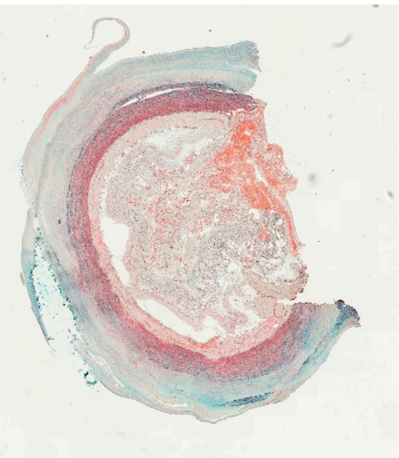
Sample 008-I

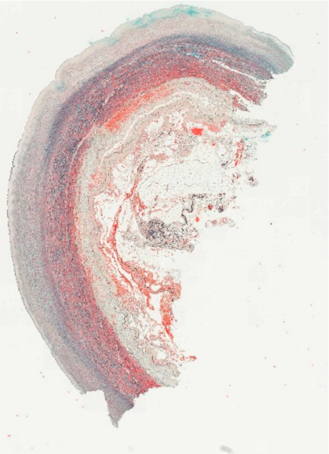
Sample 008-II

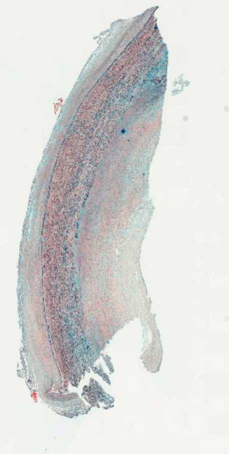
sample 008-III

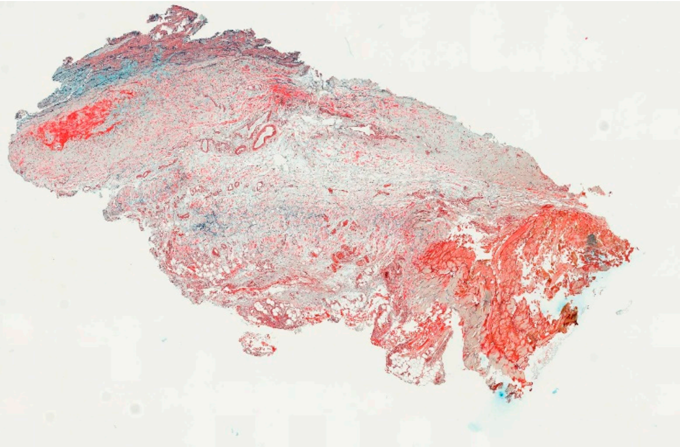
Sample 009-I

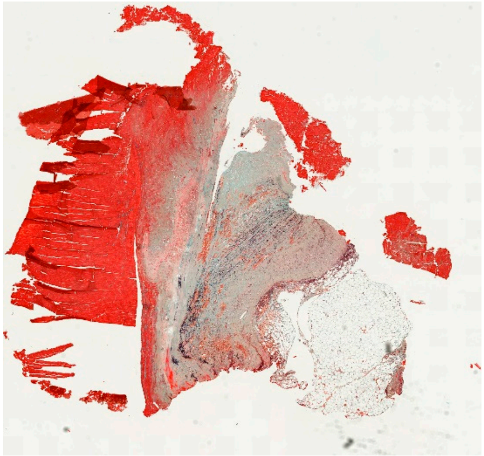
Sample 010-II

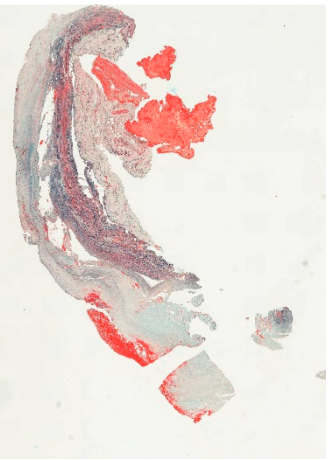

Sample 010-III

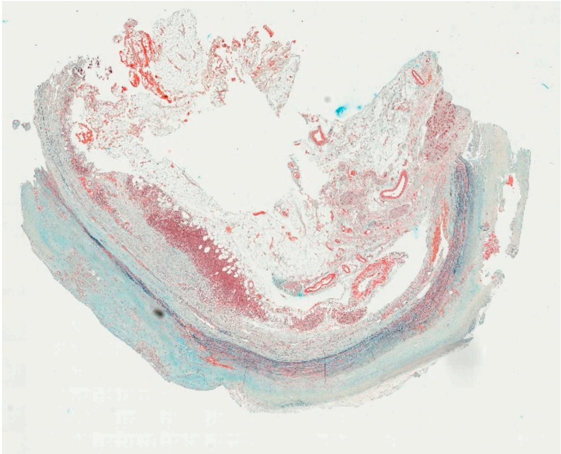

Sample 011-I

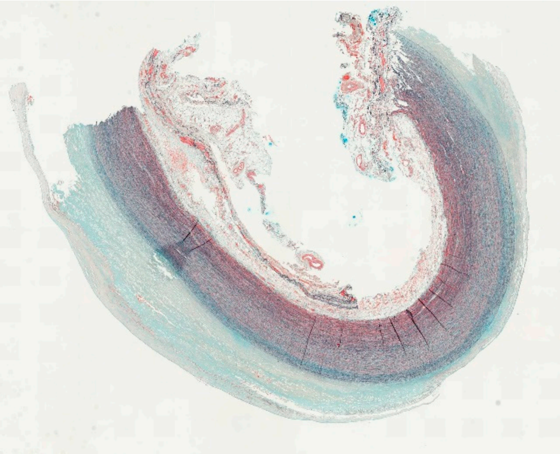

Sample 011-II

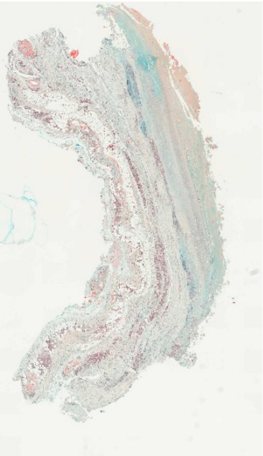
Sample 013-I

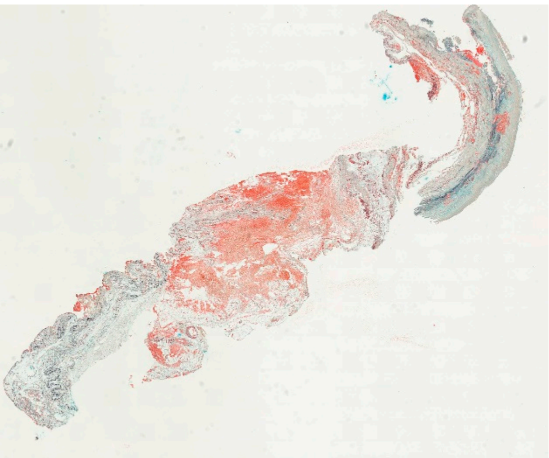
Sample 013-II

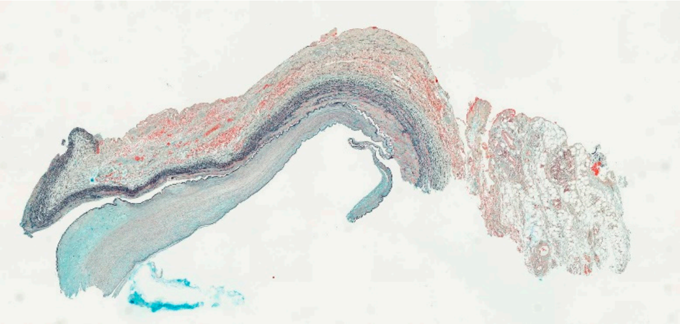
Sample 013-III

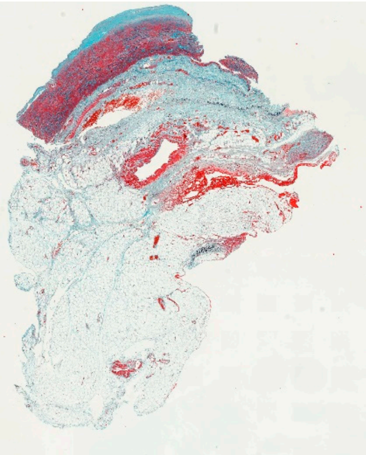
Sample 015-II

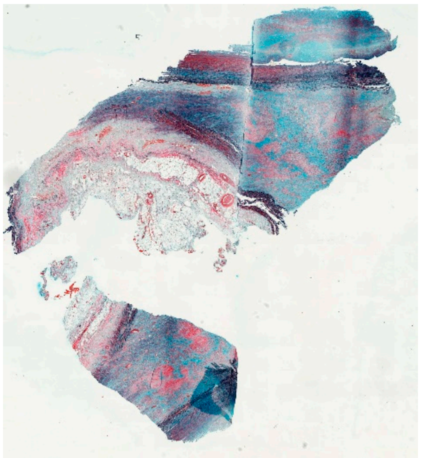

Sample 016-I

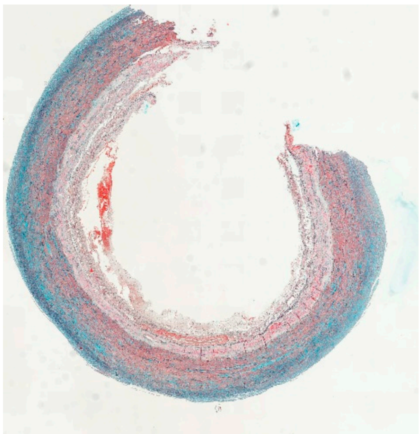

Sample 018-I

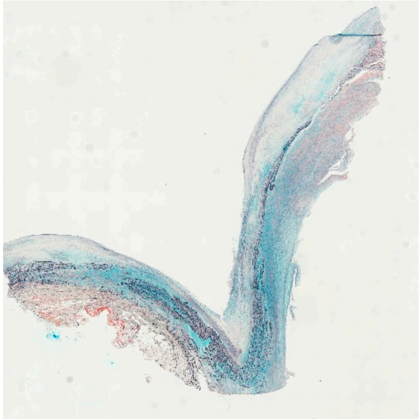

Sample 18-II

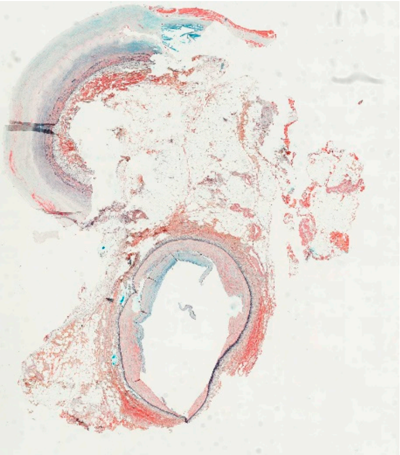
Sample 018-III

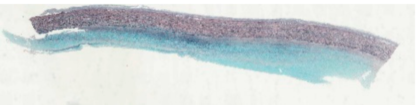
Sample 019-I-1

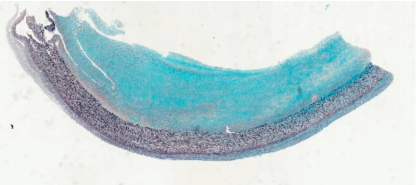
Sample 19-I-2

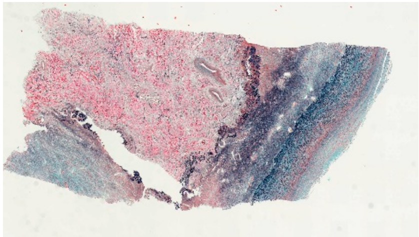
Sample 019-II

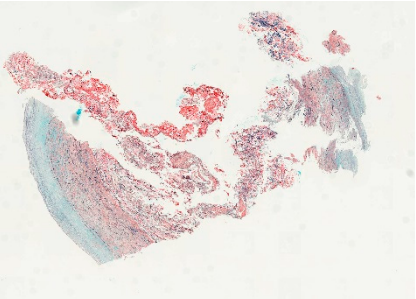
Sample 019-III

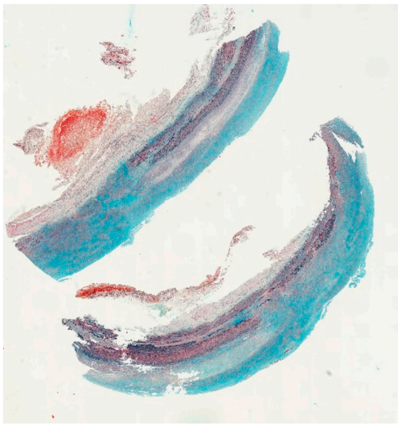

Sample 020-I

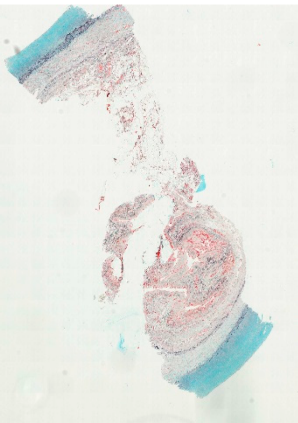

Sample 020-II

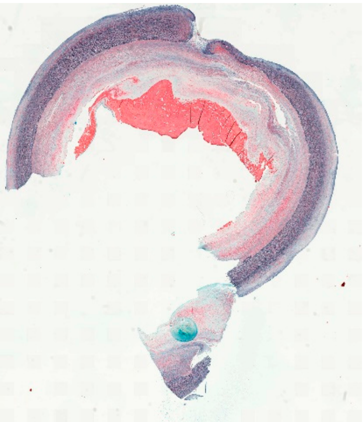

Sample 023-I

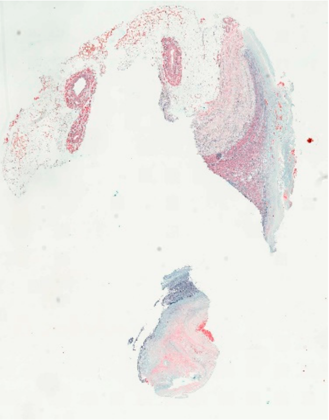

Sample 023-II

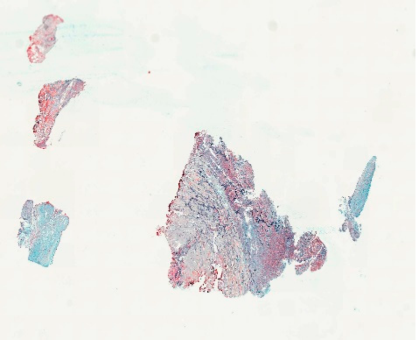
Sample 024-IV

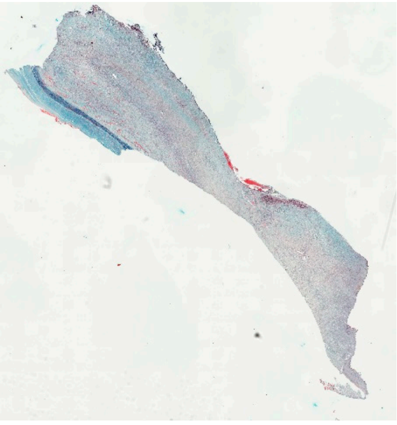
Sample 025-I

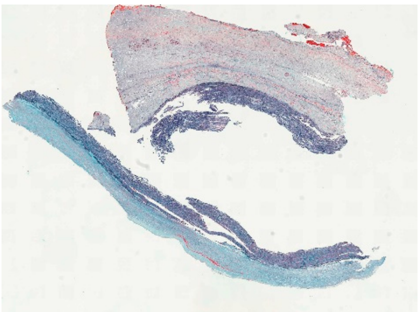
Sample 025-II

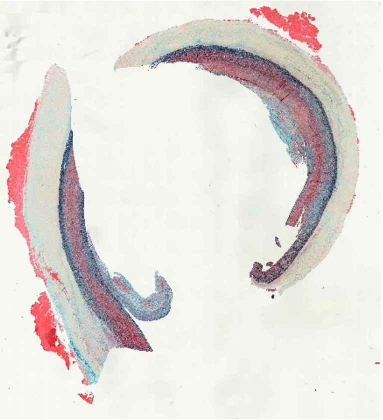
Sample 026-I

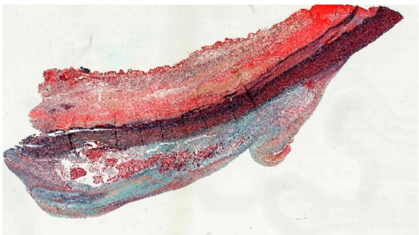
Sample 026-V

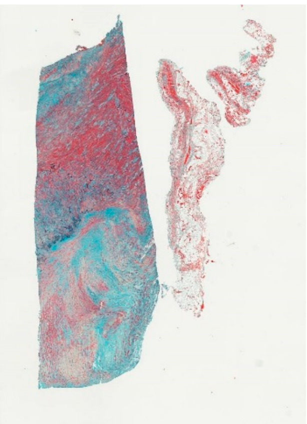
Sample 027-I

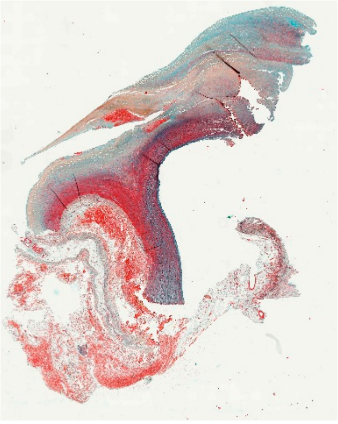
Sample 027-II

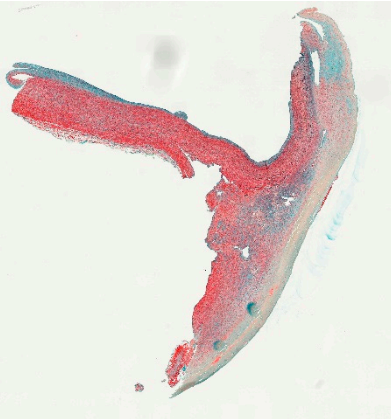
Sample 027-III

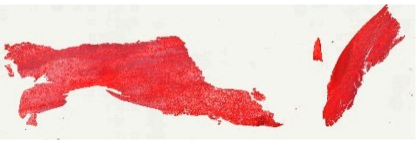
Sample 028-I

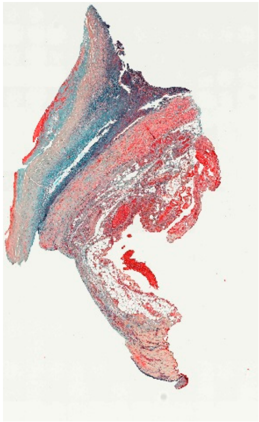

Sample 028-II

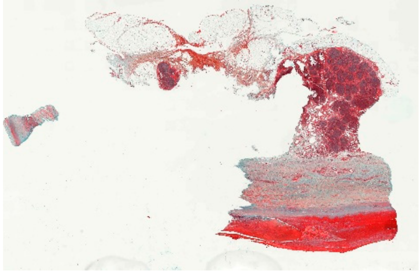

Sample 028-III

Western blot analysis

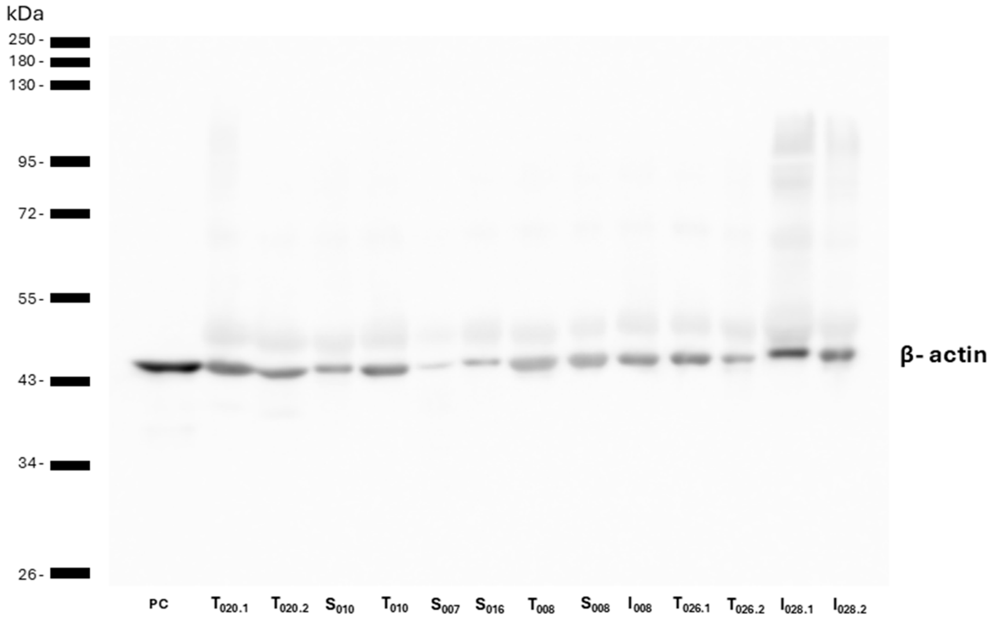

Western blot analysis 20230404_1; Protein expression of TIMP-1; PC positive control; T thoracic aortic segment; S suprarenal aortic segment, I infrarenal aortic segment

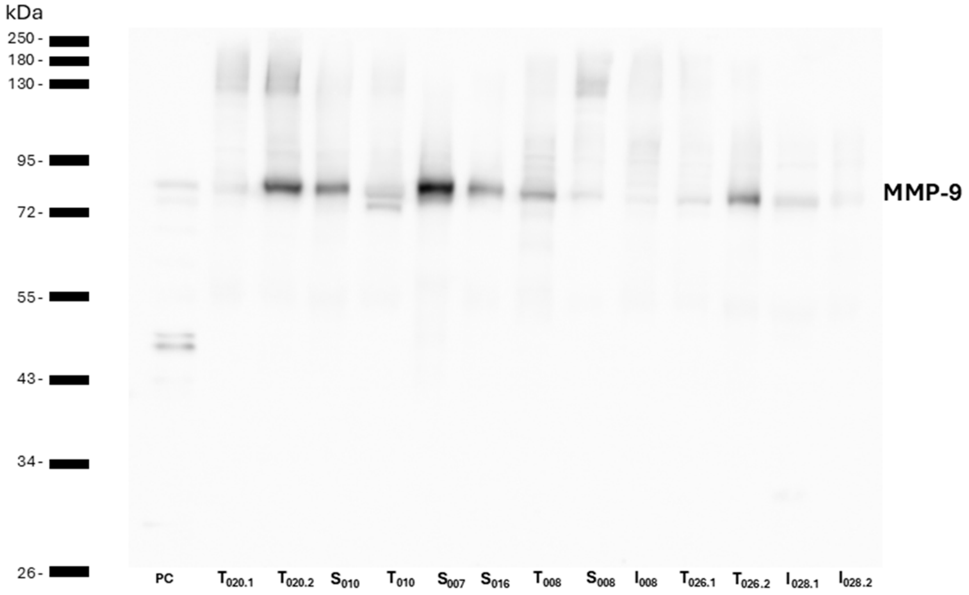

Western blot analysis 20230404_2; Protein expression of MMP-9; PC positive control; T thoracic aortic segment; S suprarenal aortic segment, I infrarenal aortic segment

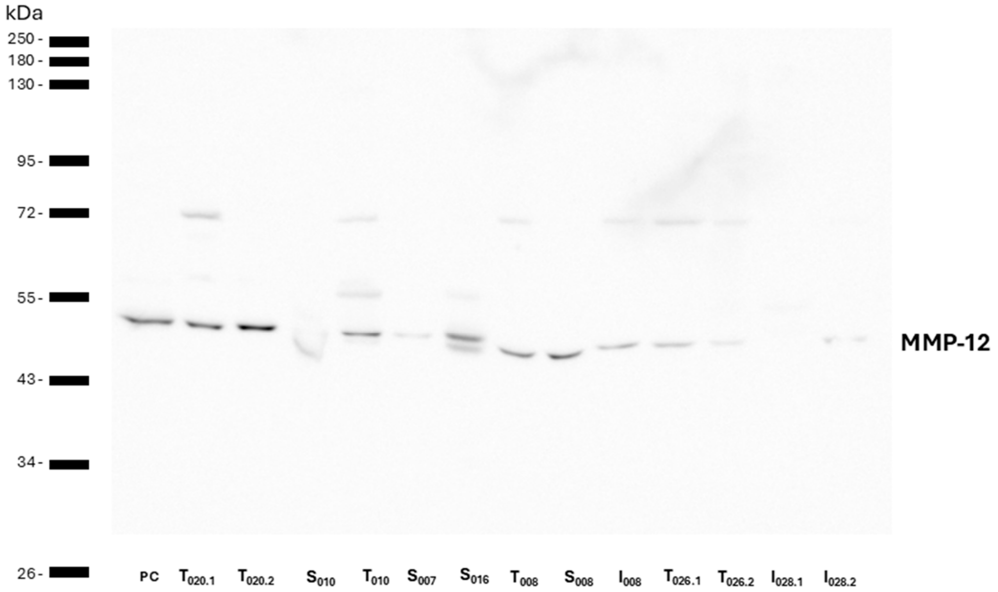

Western blot analysis 20230404_3; Protein expression of MMP-12; PC positive control; T thoracic aortic segment; S suprarenal aortic segment, I infrarenal aortic segment

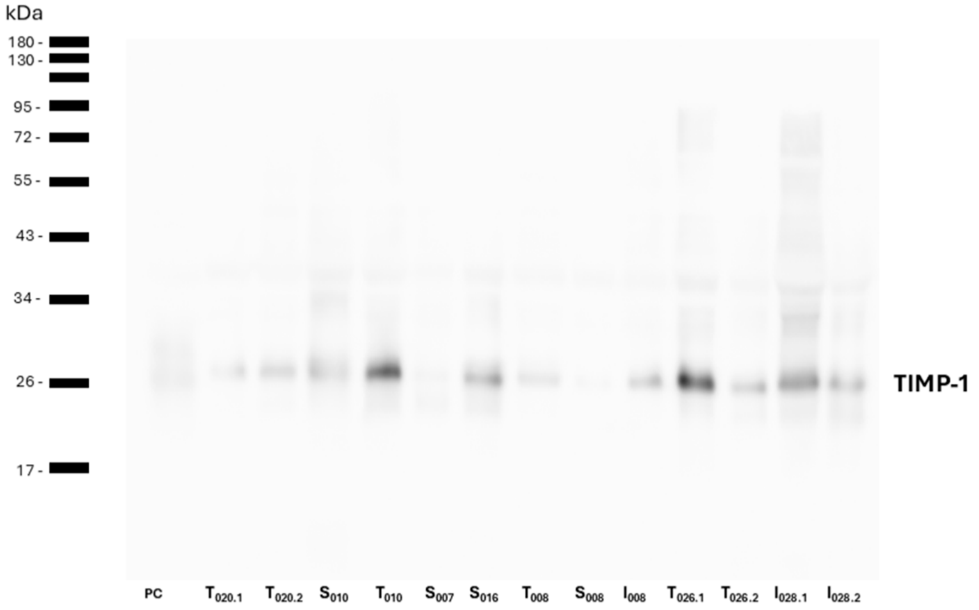

Western blot analysis 20230404_4; Protein expression of β-actin; PC positive control; T thoracic aortic segment; S suprarenal aortic segment, I infrarenal aortic segment

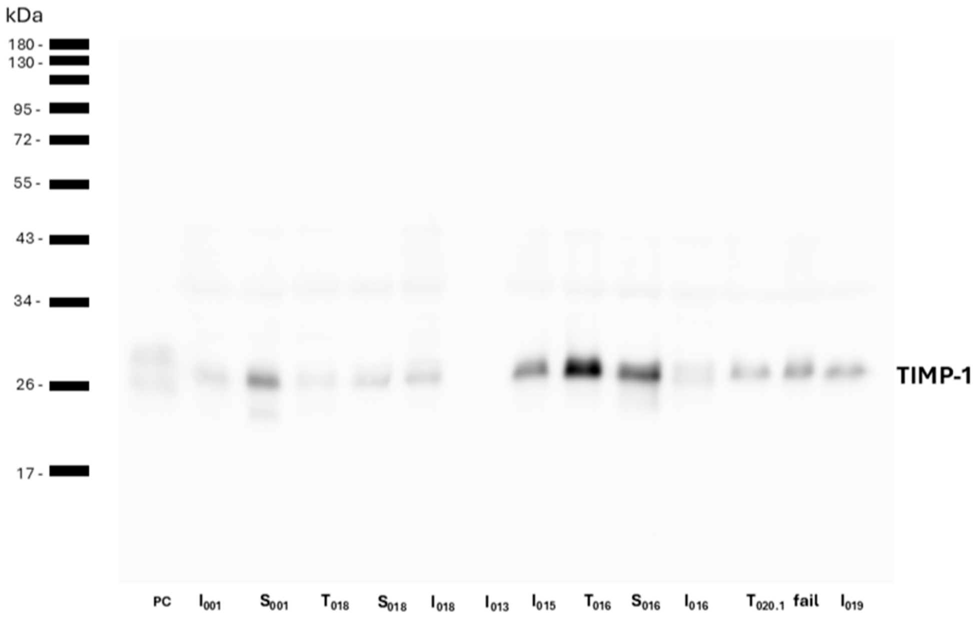

Western blot analysis 20230331_1; Protein expression TIMP-1; PC positive control; T thoracic aortic segment; S suprarenal aortic segment, I infrarenal aortic segment

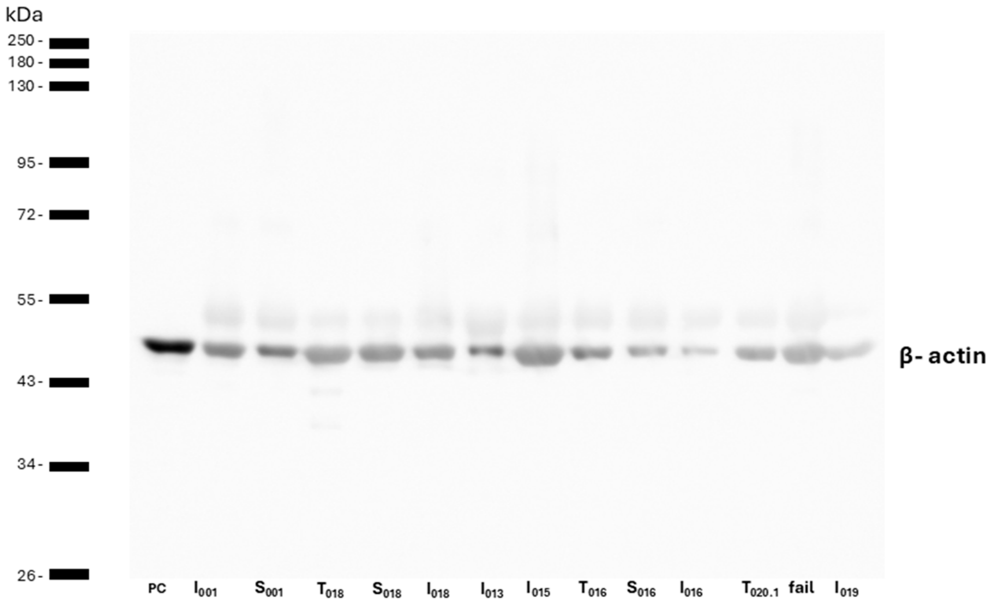

Western blot analysis 20230331_2; Protein expression of β-actin; PC positive control; T thoracic aortic segment; S suprarenal aortic segment, I infrarenal aortic segment

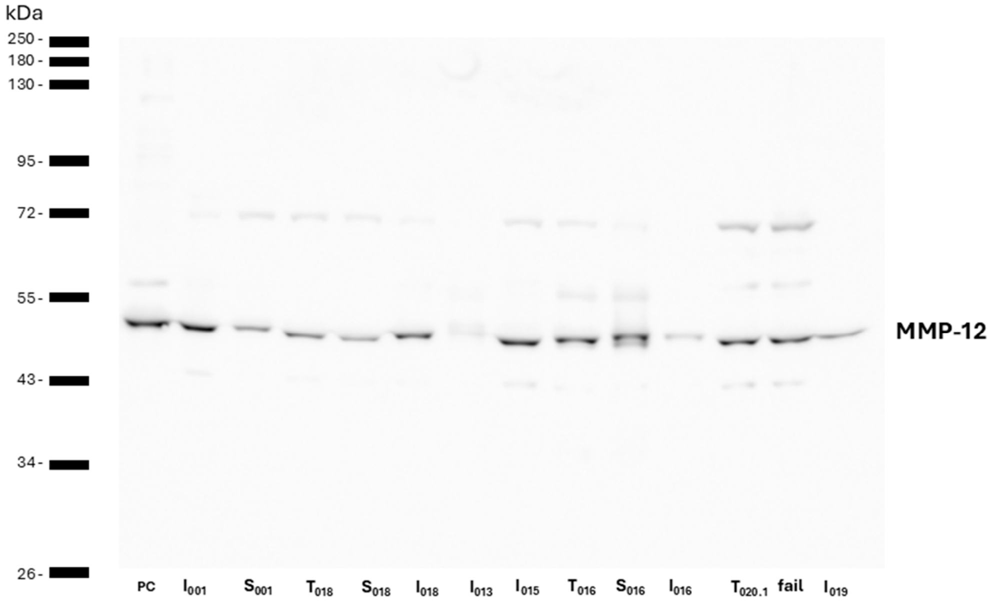

Western blot analysis 20230331_3; Protein expression of MMP-12; PC positive control; T thoracic aortic segment; S suprarenal aortic segment, I infrarenal aortic segment

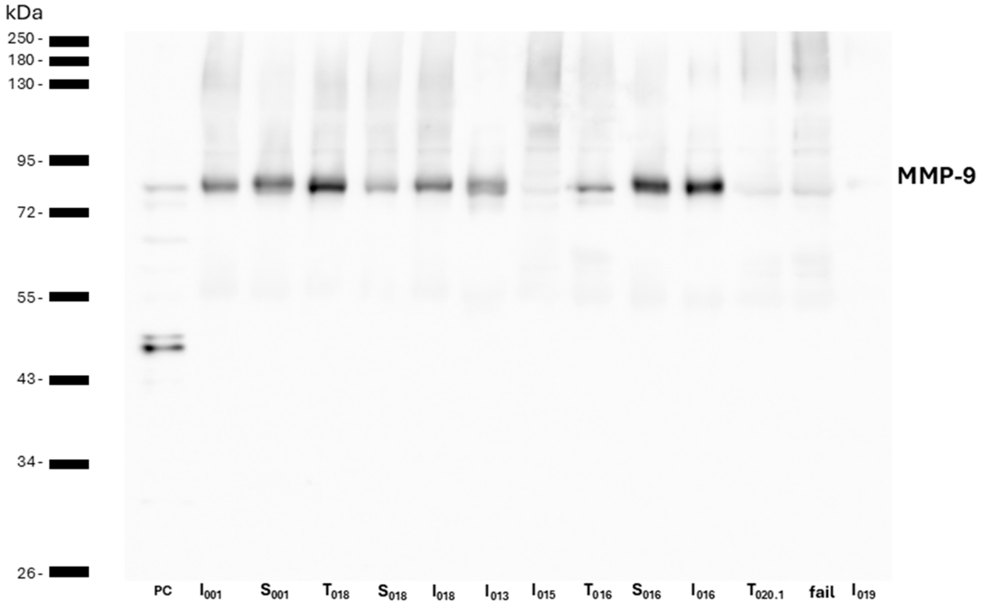

Western blot analysis 20230331_4; Protein expression of MMP-9; PC positive control; T thoracic aortic segment; S suprarenal aortic segment, I infrarenal aortic segment

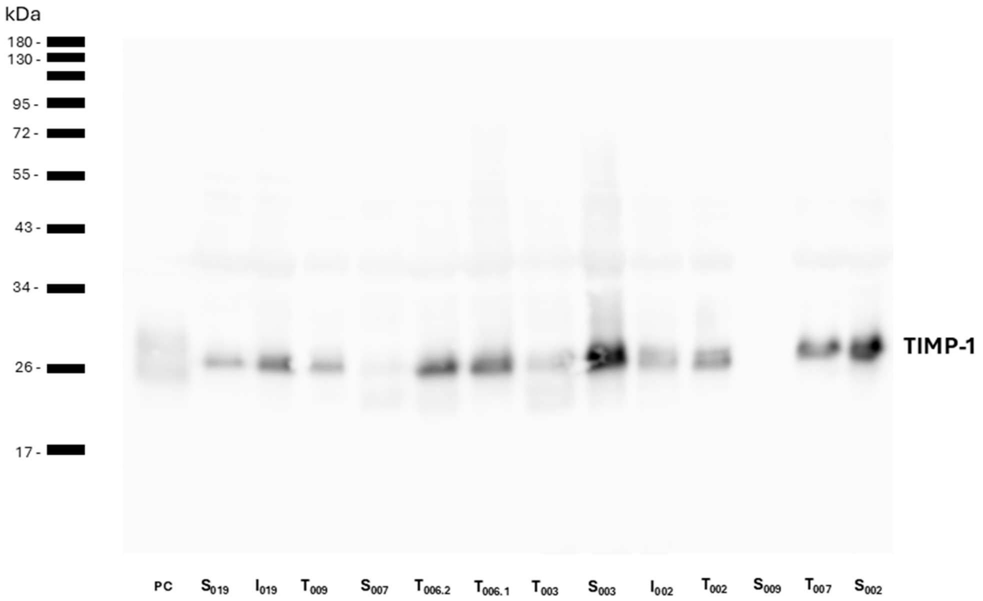

Western blot analysis 20230324_1; Protein expression of TIMP-1; PC positive control; T thoracic aortic segment; S suprarenal aortic segment, I infrarenal aortic segment

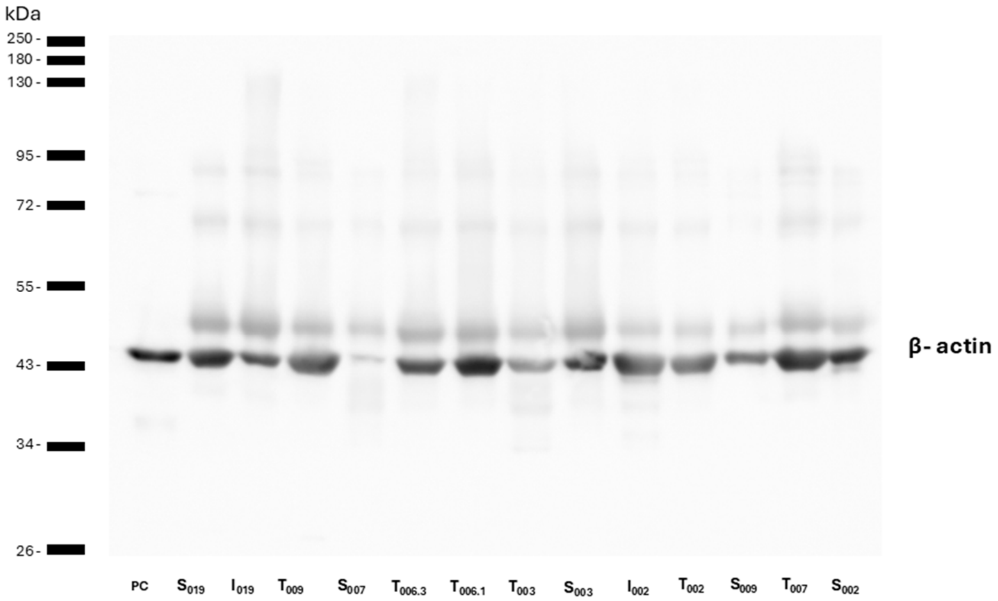

Western blot analysis 20230324_2; Protein expression of β-actin; PC positive control; T thoracic aortic segment; S suprarenal aortic segment, I infrarenal aortic segment

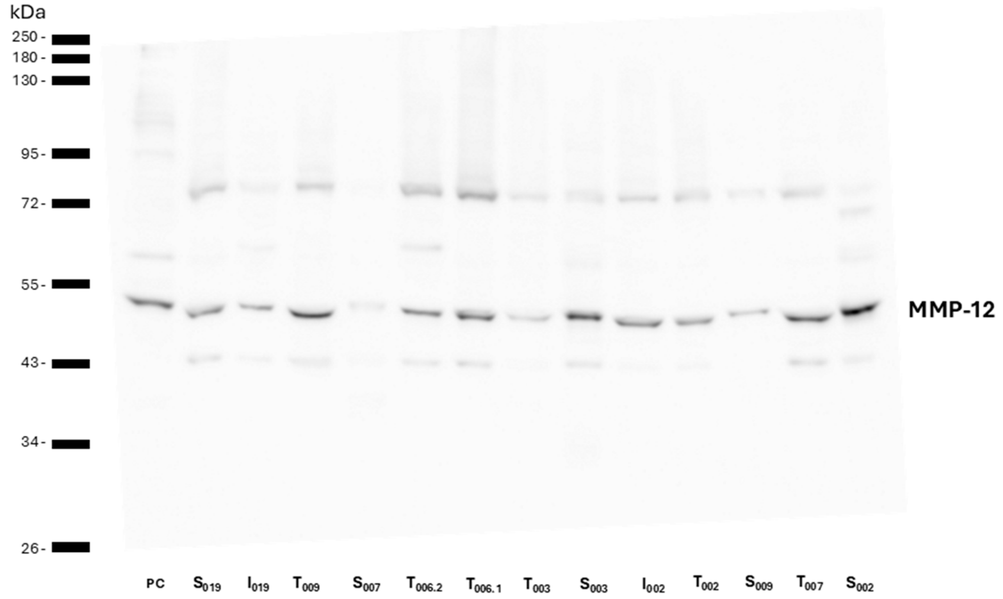

Western blot analysis 20230324_3; Protein expression of MMP-12;PC positive control; T thoracic aortic segment; S suprarenal aortic segment, I infrarenal aortic segment

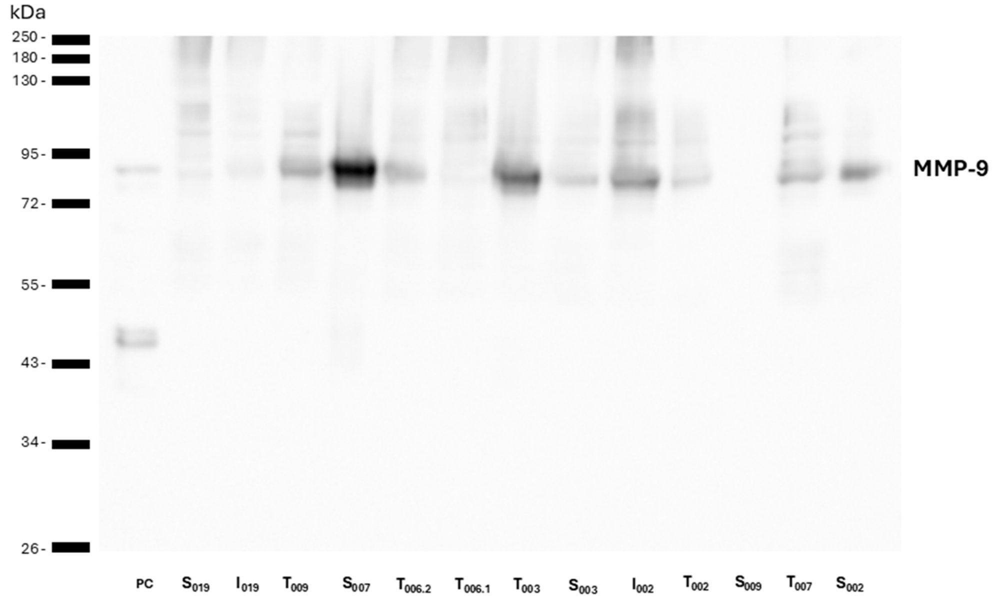

Western blot analysis 20230324_4; Protein expression of MMP-9; PC positive control; T thoracic aortic segment; S suprarenal aortic segment, I infrarenal aortic segment

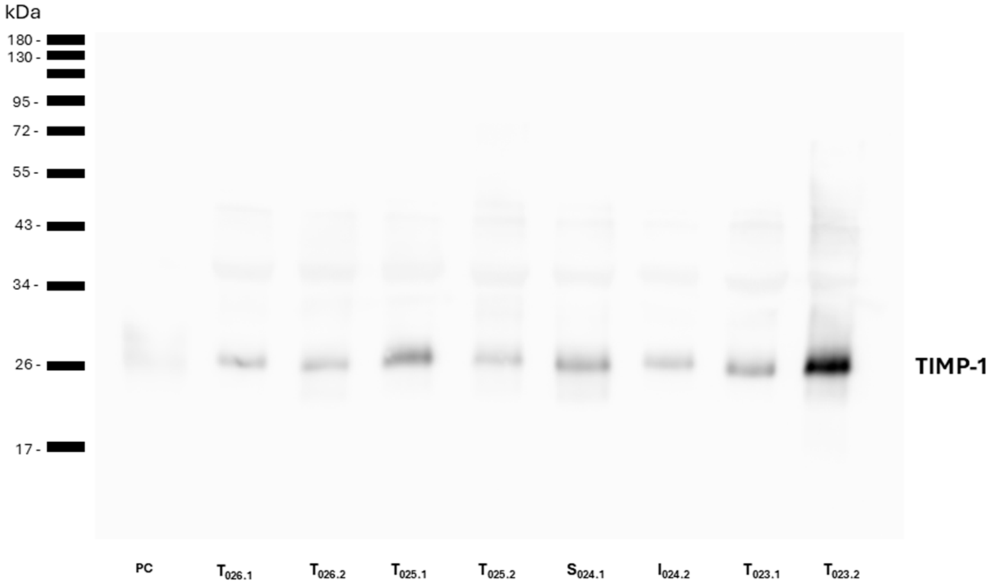

Western blot analysis 20230314_1;Protein expression of TIMP-1; PC positive control; T thoracic aortic segment; S suprarenal aortic segment, I infrarenal aortic segment

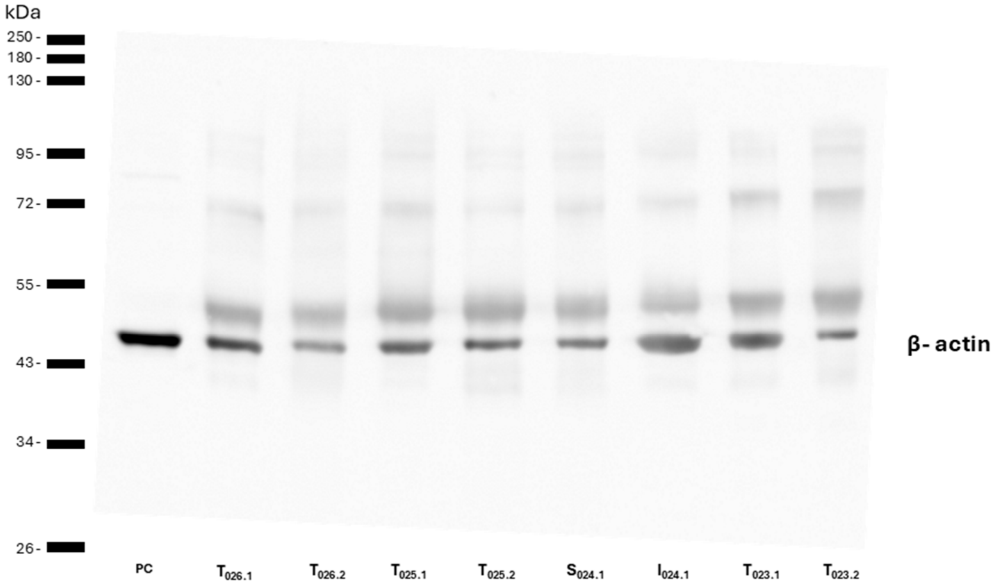

Western blot analysis 20230314_2; Protein expression of β-actin; PC positive control; T thoracic aortic segment; S suprarenal aortic segment, I infrarenal aortic segment

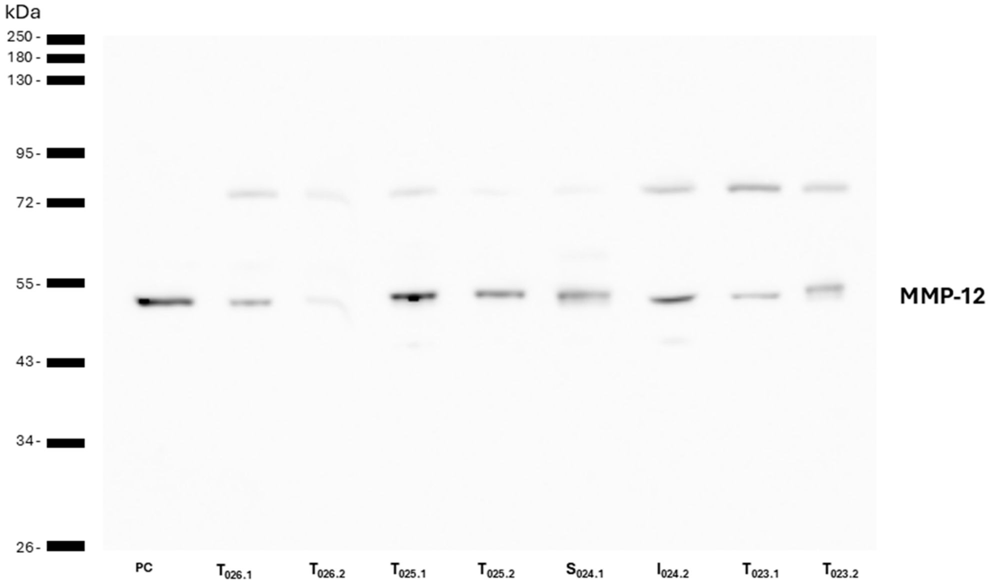

Western blot analysis 20230314_3; Protein expression of MMP-12; PC positive control; T thoracic aortic segment; S suprarenal aortic segment, I infrarenal aortic segment

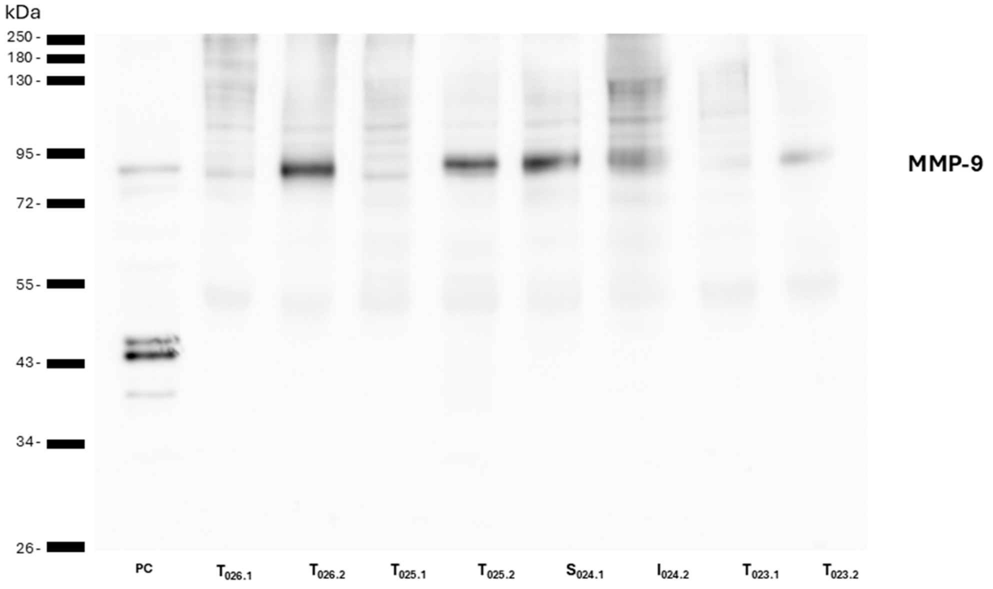

Western blot analysis 20230314_4; Protein expression of MMP-9; PC positive control; T thoracic aortic segment; S suprarenal aortic segment, I infrarenal aortic segment

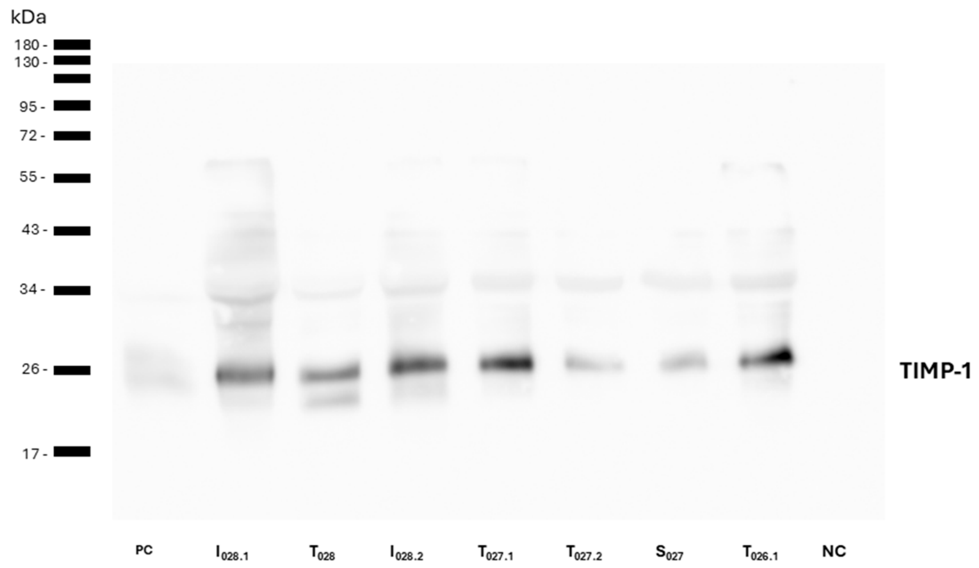

Western blot analysis 20230310_1; Protein expression of TIMP-1; PC positive control; T thoracic aortic segment; S suprarenal aortic segment, I infrarenal aortic segment; NC negative control

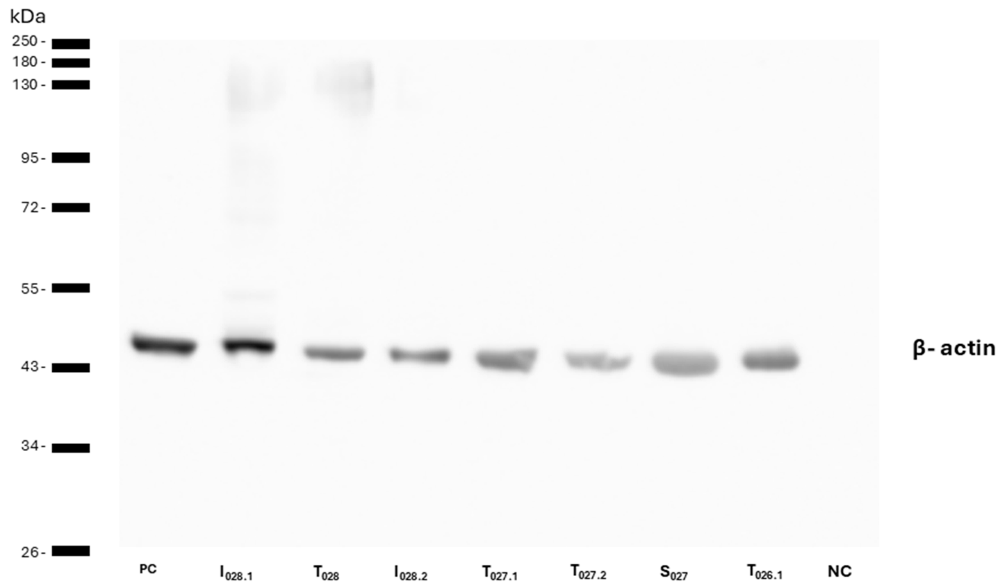

Western blot analysis 20230310_2; Protein expression of ß-actin; PC positive control; T thoracic aortic segment; S suprarenal aortic segment, I infrarenal aortic segment; NC negative control

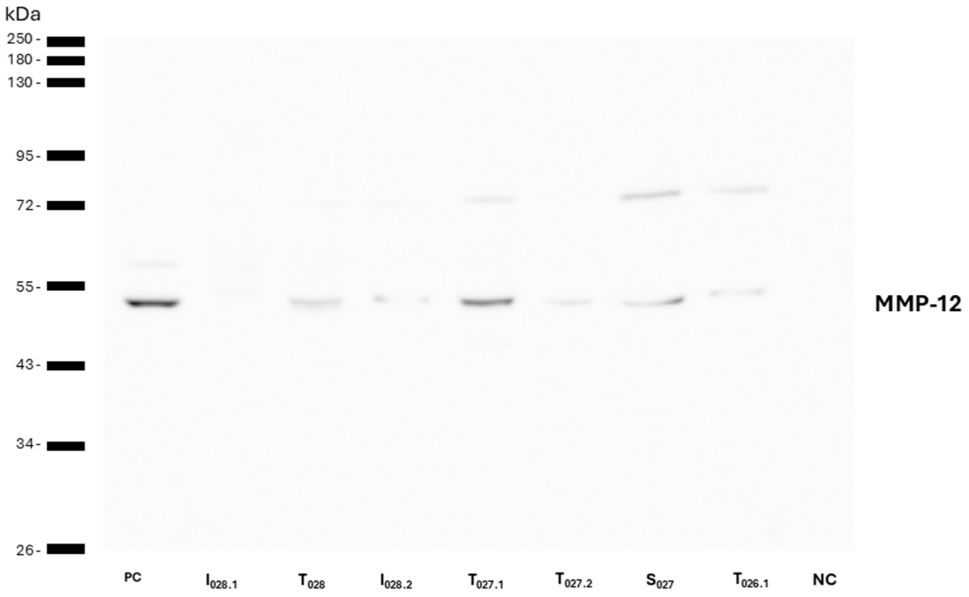

Western blot analysis 20230310_3; Protein expression of MMP-12; PC positive control; T thoracic aortic segment; S suprarenal aortic segment, I infrarenal aortic segment

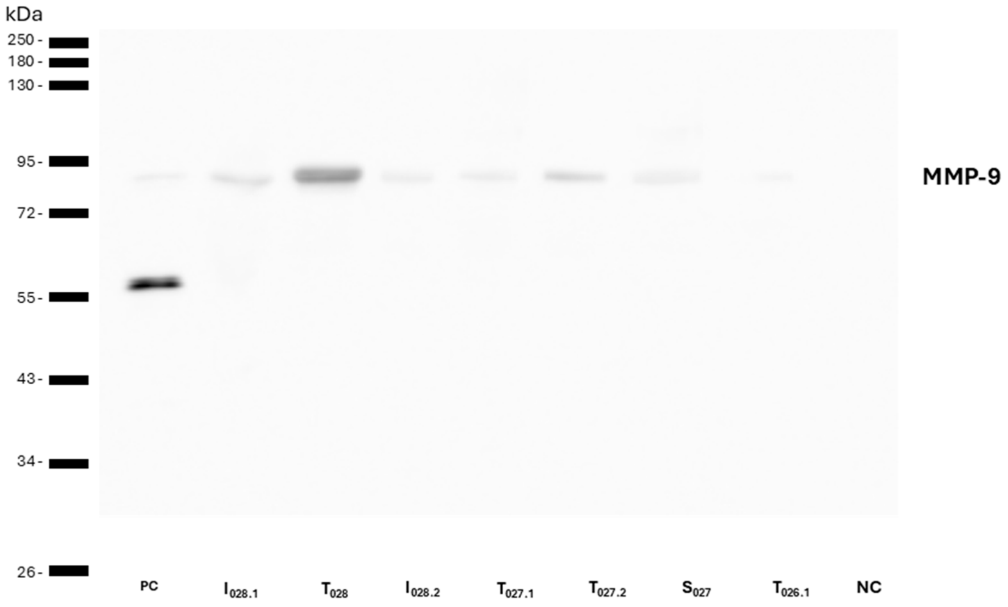

Western blot analysis 20230310_4; Protein expression of MMP-9; PC positive control; T thoracic aortic segment; S suprarenal aortic segment, I infrarenal aortic segment; NC negative control

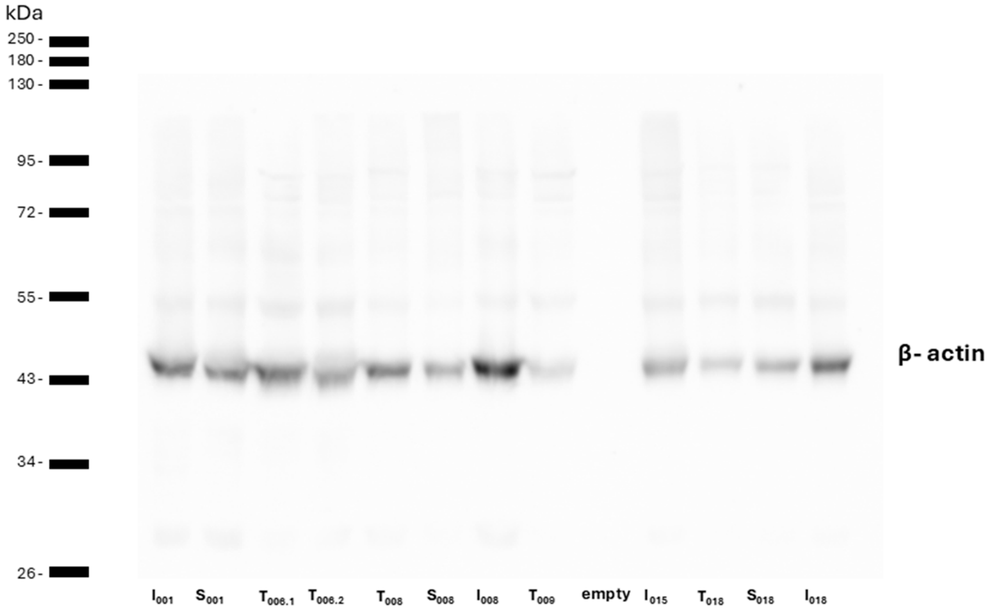

Western blot analysis 20250109_1; Protein expression of ß-actin; T thoracic aortic segment; S suprarenal aortic segment, I infrarenal aortic segment

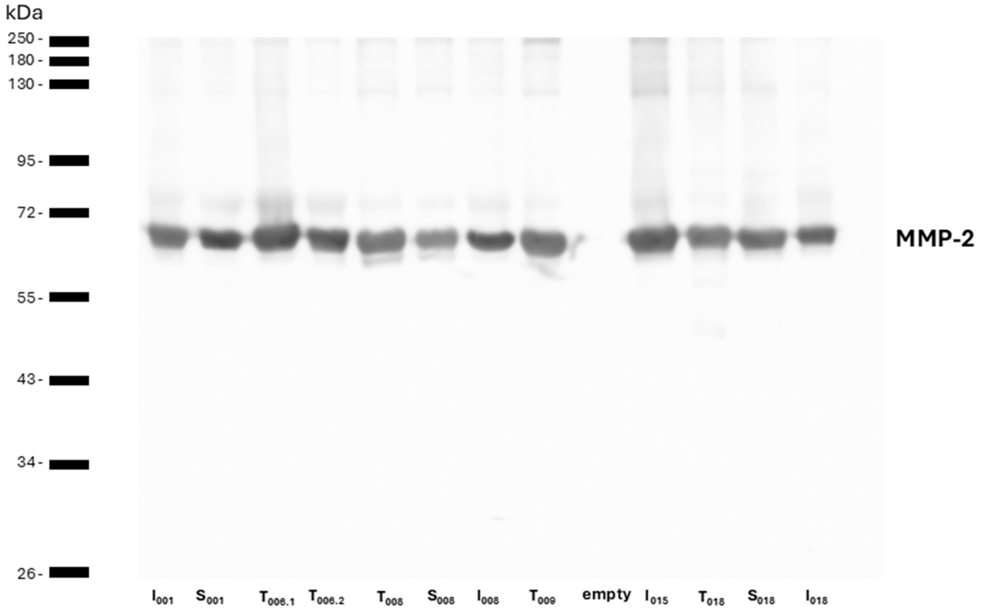

Western blot analysis 20250109_2; Protein expression of MMP-2; T thoracic aortic segment; S suprarenal aortic segment, I infrarenal aortic segment

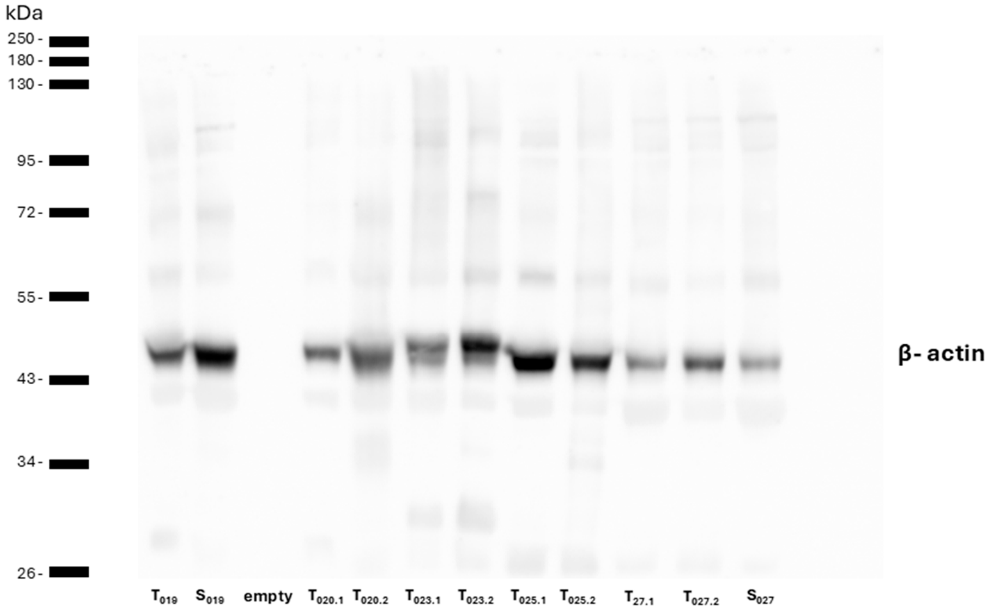

Western blot analysis 20250109_3; Protein expression of ß-actin; T thoracic aortic segment; S suprarenal aortic segment, I infrarenal aortic segment

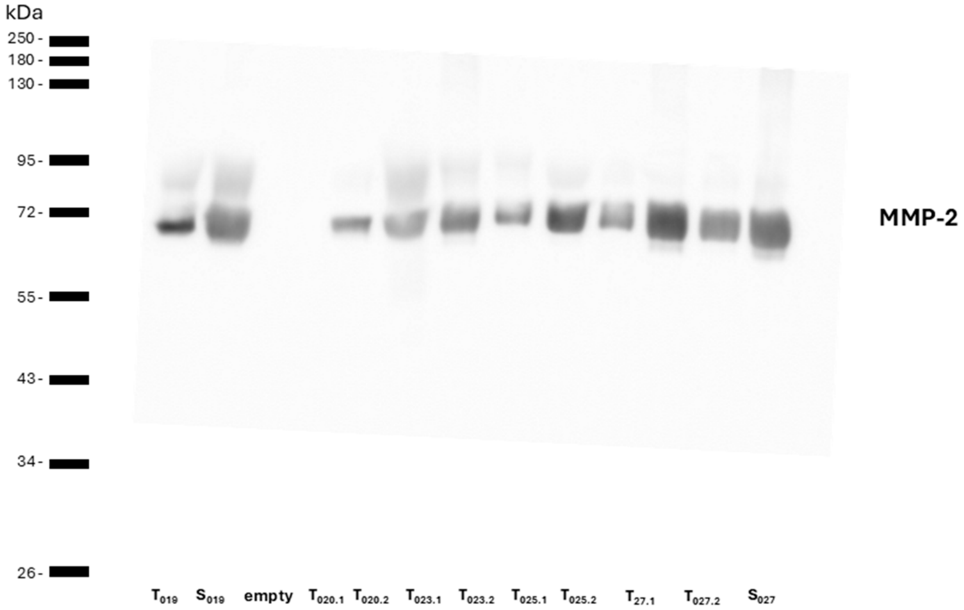

Western blot analysis 20250109_4; Protein expression of MMP-1; T thoracic aortic segment; S suprarenal aortic segment, I infrarenal aortic segment
